# Supplementary material for: A national cross-sectional survey of public perceptions of the COVID-19 pandemic: Self-reported beliefs, knowledge, and behaviors
Source: PLoS One. 2020 Oct 23;15(10):e0241259. doi: 10.1371/journal.pone.0241259 (PMC7584165; doi:10.1371/journal.pone.0241259)
Supplement: S1 Appendix — (DOCX) [file pone.0241259.s001.docx]

**S1 Appendix: Socio-Cultural Implications of COVID-19**

*Public Perceptions Survey*

**Dans quelle langue souhaitez-vous répondre à ce sondage? / In what language would you like to complete this survey?**

**O** Français

**O** English

[After language selection, survey begins with implied consent in the appropriate language then on to Q1]

**SCREENING QUESTIONS FOR QUOTA MANAGEMENT**

**YEAR / MONTH**

Dropdown

What is your date of birth?

YEAR

_1918 1918

…

_2018 2018

MONTH

_1 January

_2 February

_3 March

_4 April

_5 May

_6 June

_7 July

_8 August

_9 September

_10 October

_11 November

_12 December

RESP_AGE

Single response

**[PN: THANK AND TERMINATE IF UNDER 18]**

**S2. What was your assigned sex at birth (i.e., what does it say on your birth certificate)?**

**O** Female

**O** Male

**O** Prefer not to answer

**S3. What gender do you most identity with?**

**▢** Woman/girl

**▢** Man/boy

**▢** Non-binary

**▢** Two-spirited

**▢** Prefer to self-describe: __________

**O** Prefer not to answer

**S4. What province or territory do you live in?**

**O** British Columbia

**O** Alberta

**O** Saskatchewan

**O** Manitoba

**O** Ontario

**O** Quebec

**O** Newfoundland and Labrador

**O** Prince Edward Island

**O** New Brunswick

**O** Nova Scotia

**O** Yukon

**O** Northwest Territories

**O** Nunavut

**O** Prefer not to answer

**MAIN QUESTIONNAIRE BODY**

1. **Are you aware of the current COVID-19 pandemic?**

**O** Yes

**O** No

**[IF Q.1=”NO”, GO TO ‘READ SCREENa’ AT THE BEGINNING OF THE DEMOGRAPHIC SECTION]**

**We would like to ask you some questions about the novel coronavirus that causes COVID-19. This virus is sometimes referred to as SARS-CoV-2 (severe acute respiratory syndrome coronavirus 2).**

**We are interested in your perspectives, experiences, and understanding of COVID-19.**

**Please answer all questions without checking the internet or other sources*.***

1. **Have you had an illness that you believe was COVID-19 (currently or previously)?**

**O** Yes, and I tested positive

**O** Yes, but I was not tested

**O** Yes, but I tested negative

**O** No

**O** Don't know

**O** Prefer not to answer

1. **Do you know someone who has tested positive for COVID-19? Please select all that apply.**

**▢** Yes, family members living in the same house as me

**▢** Yes, family members not living in the same house as me

**▢** Yes, close friends

**▢** Yes, colleagues or acquaintances

**▢** No, I do not personally know anyone who has been diagnosed with COVID-19

**O** Prefer not to answer

1. **How serious of a problem is COVID-19 in Canada currently?**

| **O** | **O** | **O** | **O** | **O** | **O** |
| --- | --- | --- | --- | --- | --- |
| Not serious | Slightly serious | Somewhat serious | Moderately serious | Very serious | Prefer not to answer |

1. **How serious of a problem is COVID-19 in Canada *compared to other countries in the world?***

| **O** | **O** | **O** | **O** | **O** | **O** |
| --- | --- | --- | --- | --- | --- |
| Much less  serious | Slightly less serious | About the same | Slightly more serious | Much more serious | Prefer not to answer |

1. ***At the start of 2020*, how would you rate the following aspects of your overall health:**

**[RANDOMIZE ITEMS]**

|  | **Poor** | **Fair** | **Good** | **Very good** | **Excellent** | **Prefer not to answer** |
| --- | --- | --- | --- | --- | --- | --- |
| Mental / emotional health | **O** | **O** | **O** | **O** | **O** | **O** |
| Physical health | **O** | **O** | **O** | **O** | **O** | **O** |
| Social health | **O** | **O** | **O** | **O** | **O** | **O** |
| Economic health | **O** | **O** | **O** | **O** | **O** | **O** |
| Spiritual health | **O** | **O** | **O** | **O** | **O** | **O** |

1. ***Currently*, how would you rate the following aspects of your overall health:**

**[ASK ITEMS IN Q.7 IN THE SAME ORDER AS RANDOMIZED IN Q.6]**

|  | **Poor** | **Fair** | **Good** | **Very good** | **Excellent** | **Prefer not to answer** |
| --- | --- | --- | --- | --- | --- | --- |
| Mental / emotional health | **O** | **O** | **O** | **O** | **O** | **O** |
| Physical health | **O** | **O** | **O** | **O** | **O** | **O** |
| Social health | **O** | **O** | **O** | **O** | **O** | **O** |
| Economic health | **O** | **O** | **O** | **O** | **O** | **O** |
| Spiritual health | **O** | **O** | **O** | **O** | **O** | **O** |

1. **Please indicate how much you agree with each of the following statements:**

**[****RANDOMIZE ITEMS 8.1, 8.2 AND 8.3. ITEMS 8.4 AND 8.5 ARE ALWAYS ASKED LAST]**

|  | **Strongly Disagree** | **Disagree** | **Somewhat Disagree** | **Undecided** | **Somewhat Agree** | **Agree** | **Strongly Agree** | **Prefer not to answer/ Don’t Know** |
| --- | --- | --- | --- | --- | --- | --- | --- | --- |
| - 1. The COVID-19 pandemic makes me feel helpless | **O** | **O** | **O** | **O** | **O** | **O** | **O** | **O** |
| - 1. The COVID-19 pandemic is very stressful | **O** | **O** | **O** | **O** | **O** | **O** | **O** | **O** |
| - 1. The *federal* government is NOT doing enough to support people affected by COVID-19 | **O** | **O** | **O** | **O** | **O** | **O** | **O** | **O** |
| - 1. My *provincial* government is NOT doing enough to support people affected by COVID-19 | **O** | **O** | **O** | **O** | **O** | **O** | **O** | **O** |

1. **Please rate your degree of concern for each of the following statements.**

**[ASK 9.1 ONLY IF Q.2=”Yes, but I tested negative”, “No” or “Don’t know”]**

**[ALWAYS ASK 9.1 AND 9.2 FIRST]**

**[RANDOMIZE ITEMS 9.3, 9.4 AND 9.5]**

|  | **Not at all concerned** | **Slightly concerned** | **Somewhat concerned** | **Moderately concerned** | **Extremely concerned** | **Prefer not to answer/ Not Applicable** |
| --- | --- | --- | --- | --- | --- | --- |
| - 1. I will contract COVID-19 | **O** | **O** | **O** | **O** | **O** | **O** |
| - 1. A family member will contract COVID-19 | **O** | **O** | **O** | **O** | **O** | **O** |
| - 1. People will not be able to access the healthcare they need because of COVID-19 restrictions | **O** | **O** | **O** | **O** | **O** | **O** |
| - 1. There will not be enough protective equipment (e.g. masks) for hospital staff to stay safe | **O** | **O** | **O** | **O** | **O** | **O** |
| - 1. There will not be enough hospital equipment (e.g. beds) to care for patients with COVID-19 | **O** | **O** | **O** | **O** | **O** | **O** |

1. **What concerns you the most about the COVID-19 pandemic?**

[Open ended response to be coded]

**O** Prefer not to answer

1. **How do you rate your general understanding of how the virus that causes COVID-19 is spread?**

| **O** | **O** | **O** | **O** | **O** | **O** |
| --- | --- | --- | --- | --- | --- |
| Poor | Fair | Good | Very Good | Excellent | Prefer not to answer |

1. **Please rate how much you agree with each of the following statements about the transmission of the virus that causes COVID-19:**

**[RANDOMIZE ITEMS]**

|  | **Strongly disagree** | **Disagree** | **Some**  **what disagree** | **Neutral** | **Some**  **what agree** | **Agree** | **Strongly agree** | **Prefer not to answer/ Don’t Know** |
| --- | --- | --- | --- | --- | --- | --- | --- | --- |
| - 1. The virus can be spread through food and drink | **O** | **O** | **O** | **O** | **O** | **O** | **O** | **O** |
| - 1. People can be infected with COVID-19 but not show any symptoms | **O** | **O** | **O** | **O** | **O** | **O** | **O** | **O** |
| - 1. In order for the virus to spread, you need to have been in *close contact* with someone who has symptoms of COVID-19 | **O** | **O** | **O** | **O** | **O** | **O** | **O** | **O** |
| - 1. Wearing a face mask when out in public can help prevent the virus from spreading | **O** | **O** | **O** | **O** | **O** | **O** | **O** | **O** |
| - 1. Nothing we do will stop the spread of the virus; we are all going to get COVID-19 eventually | **O** | **O** | **O** | **O** | **O** | **O** | **O** | **O** |
| - 1. My province’s testing procedures for COVID-19 (who is tested, number of tests) are sufficient | **O** | **O** | **O** | **O** | **O** | **O** | **O** | **O** |

1. **How often do you look for information about COVID-19?**

| **O** | **O** | **O** | **O** | **O** | **O** |
| --- | --- | --- | --- | --- | --- |
| Never | Once a week | Every couple of days | Once a day | Several times a day | Prefer not to answer |

1. **What sources have you gone to for information about COVID-19 over the last 2 weeks? Please select all sources that you accessed.**

**Governmental and public health website sources**

**▢**Canadian national websites

**▢**Canadian provincial websites

**▢**American government websites

**▢**The World Health Organization’s website

**Television, print, or website sources**

**▢**Canadian television news

**▢**American television news

**▢**Canadian newspapers/magazines

**▢**American newspapers/ magazine

**▢**Canadian news websites

**▢**American news websites

**▢**International news sources including websites, broadcasts, and newspapers

**▢**Other Websites

**Social media sources**

**▢** Posts on social media from *health organizations and government*

**▢**Posts on social media from *friends and family*

**▢**Posts on social media from *influencers or celebrities*

**Word-of-Mouth sources**

**▢**Conversations with friends and family

**▢**Conversations with work colleagues

**▢**Conversations with acquaintances or individuals outside my circle

**▢**Conversations with a healthcare provider

**▢** Other: **[PLEASE SPECIFY]**__________

**O** I have not gone to any source for information about COVID-19 during the past 2 weeks.

**O** None of the above

**O** Prefer not to answer

1. **What kind of information did you look for during the last 2 weeks related to the COVID-19 pandemic? Please select all that apply.**

**▢** Symptoms of COVID-19

**▢** Infection rates

**▢** Testing rates and procedures

**▢** Personal stories from others related to COVID-19

**▢** Personal stories from those who had/have COVID-19

**▢** Progress on development of a COVID-19 vaccine and potential vaccine safety

**▢** Treatments for COVID-19 currently in use or in development

**▢** How I can personally prevent spread of the disease

**▢** Access to social services or resources

**▢** Caring for a person who is at risk

**▢** Information on my children’s education

**▢** Travel restrictions

**▢** Other: **[PLEASE SPECIFY]**__________

**O** I did not look for any information related to COVID-19 the last two weeks

**O** None of the above topics

**O** Prefer not to answer

**[PN: PLEASE USE DRAG AND DROP FOR Q.16]**

1. **Please select and rank the 3 MOST trustworthy sources for COVID-19 information and the 3 LEAST trustworthy sources for COVID-19 information.**

**Governmental and public health sources**

**▢**Canadian national websites

**▢**Canadian provincial websites

**▢**American government websites

**▢**The World Health Organization’s website

**Television, print, or online sources**

**▢**Canadian television news

**▢**American television news

**▢**Canadian newspapers/magazines

**▢**American newspapers/ magazine

**▢**Canadian news websites

**▢**American news websites

**▢**International news sources including websites, broadcasts, and newspapers

**▢**Other Websites

**Social media sources**

**▢** Posts on social media from *health organizations and government*

**▢**Posts on social media from *friends and family*

**▢**Posts on social media from *influencers or celebrities*

**Word-of-Mouth**

**▢**Conversations with friends and family

**▢**Conversations with work colleagues

**▢**Conversations with acquaintances or individuals outside my circle

**▢**Conversations with a healthcare provider

**▢** Other: **[PLEASE SPECIFY]**__________

**O** There are no trustworthy sources

**O** None of the above sources

**O** Prefer not to answer

1. **How confident are you that *you* can identify incorrect or misleading information about COVID-19?**

| **O** | **O** | **O** | **O** | **O** | **O** |
| --- | --- | --- | --- | --- | --- |
| Not at all confident | Slightly confident | Somewhat confident | Moderately confident | Extremely confident | Prefer not to answer |

1. **How confident are you that *the average person* can identify incorrect or misleading information about COVID-19?**

| **O** | **O** | **O** | **O** | **O** | **O** |
| --- | --- | --- | --- | --- | --- |
| Not at all confident | Slightly confident | Somewhat confident | Moderately confident | Extremely confident | Prefer not to answer |

1. **Have you seen or heard incorrect or misleading information about COVID-19 in the last 2 weeks? Please select all topics that apply.**

**▢** Symptoms of COVID-19

**▢** Infection rates

**▢** Testing rates and procedures

**▢** Personal stories from others related to COVID-19

**▢** Personal stories from those who had/have COVID-19

**▢** Progress on development of a COVID-19 vaccine and potential vaccine safety

**▢** Treatments for COVID-19 currently in use or in development

**▢** How I can personally prevent spread of the disease

**▢** Access to social services or resources

**▢** Caring for a person who is at risk

**▢** Information on my children’s education

**▢** Travel restrictions

**▢** Other: **[PLEASE SPECIFY]**_________________________

**O** I did not see or hear any incorrect or misleading information related to COVID-19 the last 2 weeks

**O** Prefer not to answer

**[ASK Q.19.1 IF Q.19 IS NOT “I did not see or hear any incorrect or misleading information related to COVID-19 the last 2 weeks” or “Prefer not to answer”]**

- 1. **And where did you see or hear any incorrect or misleading information? Please select all that apply.**

**Governmental and public health website sources**

**▢**Canadian national websites

**▢**Canadian provincial websites

**▢**American government websites

**▢**The World Health Organization’s website

**Television, print, or website sources**

**▢**Canadian television news

**▢**American television news

**▢**Canadian newspapers/magazines

**▢**American newspapers/ magazine

**▢**Canadian news websites

**▢**American news websites

**▢**International news sources including websites, broadcasts, and newspapers

**▢**Other Websites

**Social media sources**

**▢** Posts on social media from *health organizations and government*

**▢**Posts on social media from *friends and family*

**▢**Posts on social media from *influencers or celebrities*

**Word-of-Mouth**

**▢**Conversations with friends and family

**▢**Conversations with work colleagues

**▢**Conversations with acquaintances or individuals outside my circle

**▢**Conversations with a healthcare provider

**▢** Other: **[PLEASE SPECIFY]**__________

**O** None of the above sources

**O** I don’t remember

**O** Prefer not to answer

1. **How do you verify or check the information that you see or hear? Please select all that apply.**

**▢** Use an online search engine (e.g., Google, Yahoo)

**▢** Go directly to an online news source

**▢** Go directly to a government or health authority source

**▢** Look for scientific articles

**▢** Ask a family member or friend

**▢** Ask a medical health professional

**▢** Ask on social media about the credibility of the information

**▢** Use Snopes, or another fact-checking service

**▢** I do not use any of the above strategies

**▢** Other: **[PLEASE SPECIFY]**__________

**O** Nothing, I just choose to not believe it

**O** Nothing, I just choose to believe it

**O** Prefer not to answer

1. **Please indicate how much you agree with each of the following statements:**

**[RANDOMIZE ITEMS]**

|  | **Strongly disagree** | **Disagree** | **Some-**  **what Disagree** | **Neutral** | **Some-**  **what Agree** | **Agree** | **Strongly agree** | **Prefer not to answer/ Don’t know** |
| --- | --- | --- | --- | --- | --- | --- | --- | --- |
| - 1. I am able to find the kind of information I want about COVID-19 | **O** | **O** | **O** | **O** | **O** | **O** | **O** | **O** |
| - 1. I find it hard to determine if an information source is trustworthy or not. | **O** | **O** | **O** | **O** | **O** | **O** | **O** | **O** |

1. **Are you currently in self-isolation?**

*Self-isolation is when you are separating yourself from others, including those within your home, with the purpose of preventing the spread of the virus (whether diagnosis or undiagnosed, with or without symptoms). You do not leave the house, go to work, or get groceries or access other essential services*

**O** Yes, I am currently in self-isolation

**O** No, I am not currently in self-isolation

**O** Prefer not to answer

**[ASK Q.23 IF Q.22 = “No, I am not currently in self-isolation” or “Prefer not to answer”]**

1. **How often are you practicing social or physical distancing?**

*Social or physical distancing refers to limiting your time in spaces occupied by others, including reducing trips to visit others in person and reducing time spent in public spaces.*

*The World Health Organization has recommended that ‘physical distancing’ is the most appropriate term to use to describe these social measures. As a result, for the rest of the survey, we will use physical distancing.*

| **O** | **O** | **O** | **O** | **O** | **O** |
| --- | --- | --- | --- | --- | --- |
| Not at all | Rarely | Sometimes | Often | Always | Prefer not to answer |

**[ASK Q.24 IF Q.22 = “Yes, I am currently in self-isolation”]**

1. **Why have you been practicing self-isolation? Please select up to 3 of your top reasons.**

**▢** To protect myself

**▢** To protect other people I live with

**▢** To protect other people that I live with who are vulnerable/high risk

**▢** To protect other family members or friends that I do not live with

**▢** To protect other members of the general public

**▢** To help decrease the burden on the healthcare system

**▢** To avoid possibly receiving a fine for not following guidelines.

**▢** Other: **[PLEASE SPECIFY]**__________

**O** Prefer not to answer

**[ASK Q.25 IF Q.23 = “Sometimes”, “Often” or “Always”]**

1. **Why have you been practicing physical distancing? Please select up to 3 of your top reasons.**

**▢** To protect myself

**▢** To protect other people I live with

**▢** To protect other people that I live with who are vulnerable/high risk

**▢** To protect other family members or friends that I do not live with

**▢** To protect other members of the general public

**▢** To help decrease the burden on the healthcare system

**▢** To avoid possibly receiving a fine for not following guidelines.

**▢** Other: **[PLEASE SPECIFY]**__________

**O** Prefer not to answer

**[ASK Q.26 IF Q.23 = “Not at all” or “Rarely”]**

1. **Why are you not regularly practicing physical distancing? Please select all that apply.**

**▢** I am healthy

**▢** I am not concerned about getting the virus that causes COVID-19

**▢** I think the situation is not as bad as people are saying it is

**▢** It is important for me to continue to visit friends and/or family member during the pandemic

**▢** My work makes it very difficult to practice distancing

**▢** Everyone will get the virus that causes COVID-19 eventually, so it does not matter.

**▢** Other: **[PLEASE SPECIFY]**__________

**O** Prefer not to answer

**[ASK Q.27 IF Q.24 = “To protect other people that I live with who are vulnerable” OR IF Q.25 = “To protect other people that I live with who are vulnerable”]**

1. **You indicated that you are practicing self-isolation or social or physical distancing to protect someone who is vulnerable/high-risk. Please describe the vulnerable people you are protecting by practicing self-isolation or physical distancing, and describe the reason they are vulnerable.**

[Open ended response to be coded]

**O** Prefer not to answer

**[If Q.22 = “Yes, I am currently in self-isolation”, include READ SCREEN below]**

**[READ SCREEN]**

Social or physical distancing refers to limiting your time in spaces occupied by others, including reducing trips to visit others in person and reducing time spent in public spaces. The World Health Organization has recommended that ‘physical distancing’ is the most appropriate term to use to describe these social measures. As a result, for the rest of the survey, we will use physical distancing.

**[ASK ALL]**

1. **Which age group do you think is practicing physical distancing most consistently**?

**O** Teenagers

**O** Young adults

**O** Middle-aged adults

**O** Seniors

**O** Don’t know

**O** Prefer not to answer

1. **Which age group do you think is practicing physical distancing least consistently**?

**O** Teenagers

**O** Young adults

**O** Middle-aged adults

**O** Seniors

**O** Don’t know

**O** Prefer not to answer

1. **How much longer do you believe you can reasonably sustain your *current* level of physical distancing?**

**O** Not any longer

**O** 2 more weeks

**O** 1 more month

**O** 2 more months

**O** 3 more months

**O** 6 more months

**O** Longer than six months (as long as it is needed)

**O** Don’t know

**O** Prefer not to answer

1. **Please indicate how much you agree with each of the following statements:**

**[RANDOMIZE ITEMS]**

|  | **Strongly disagree** | **Disagree** | **Some-**  **what disagree** | **Neutral** | **Some-**  **what agree** | **Agree** | **Strongly agree** | **Prefer not to answer / Don’t know** |
| --- | --- | --- | --- | --- | --- | --- | --- | --- |
| - 1. With changes to my behaviour, I am doing a good job at preventing the spread of the virus that causes COVID-19 | **O** | **O** | **O** | **O** | **O** | **O** | **O** | **O** |
| - 1. I am doing a better job than most Canadians changing my behaviour to prevent the spread of the virus that causes COVID-19 | **O** | **O** | **O** | **O** | **O** | **O** | **O** | **O** |
| - 1. I will get vaccinated for the virus when it is developed | **O** | **O** | **O** | **O** | **O** | **O** | **O** | **O** |

**You have reached the end of this portion of the survey. Thank you for your responses!**

**Demographics**

**[READ SCREENa IF Q1=NO]**

There is a pandemic of respiratory disease caused by the novel coronavirus, also called COVID-19. This virus was first discovered in Wuhan, Hubei province in China and has since spread across the world. There are hundreds of thousands of confirmed cases and many deaths related to COVID-19, including in Canada. Please visit the [Government of Canada COVID-19 Website](https://www.canada.ca/en/public-health/services/diseases/2019-novel-coronavirus-infection.html?&utm_campaign=gc-hc-sc-coronaviruspublicedu2021-2021-0001-9762248618&utm_medium=search&utm_source=google-ads-99837326356&utm_content=text-en-428935858525&utm_term=covid) for information.

**[READ SCREEN FOR ALL]**

**We would now like to ask you some demographic questions to help categorize the responses. As a reminder, your answers will only be used in grouped data analysis – we will not be able to identify you.**

**D1. What is your current marital status?**

**O** Single, never married

**O** In a relationship, but not living together

**O** Living with partner

**O** Married

**O** Separated or divorced (in process or finalized)

**O** Widowed

**O** Prefer not to answer

**D2. What is the size of the town or city you live in?**

**O** An unincorporated area (e.g. township, village – up to 1,000 people)

**O** Small town or village (up to 5,000 people)

**O** Small city (up to 10,000 people)

**O** Medium sized city (over 10,000 people up to 100,000 people)

**O** Large city (over 100,000 people up to 1,000,000 people)

**O** Large metropolitan area (over 1,000,000 people)

**O** Don’t know

**O** Prefer not to answer

**D3. How many people live in your household, including yourself?**

*(Please include all other family members and / or roommates living in the same house)*

___ person / people [verification: integer]

**O** Prefer not to answer

**D4. How many children (under the age of 18) live in your household?** Please do not include children who are away at school. *(If you do not have any children in a specific age range, please enter ‘0’)*

**O** I do not have any children under the age of 18 years living at home

___ infants(s) (<=1 years) [verification: integer]

___ toddler(s) (1- 2 years) [verification: integer]

___ child(ren) (3 -12 years) [verification: integer]

___ teenager(s) (13-17 years) [verification: integer]

**O** Prefer not to answer

**D5. What is your current employment status? Please select all that apply.**

**▢** Employed (working full-time hours)

**▢** Employed (working part-time/casual hours)

**▢** Self-employed (working full-time hours)

**▢** Self-employed (working part-time hours)

**▢** Retired

**▢** Student (full or part-time)

**▢** Full time parent or homemaker

**▢** Military (full or part-time)

**▢** Currently unemployed or unable to work for any reason (including laid off)

**O** Prefer not to answer

**[ASK QD6 IF D5 = “Currently unemployed or unable to work for any reason (including laid off)”]**

D6. **Is your current unemployment a direct result of the COVID-19 pandemic (e.g. laid off by a company, ordered to discontinue service from the government)?**

**O** Yes

**O** No

**O** Unsure

**O** Prefer not to answer

**[ASK D7 IF D5 = “Employed (working full-time hours)”, “Employed (working part-time/casual hours”, “Self-employed (working full-time hours)”, “Self-employed (working part-time hours)”, “Military (full or part-time)” or “Currently unemployed or unable to work for any reason (including laid off)”]**

**D7. What type of industry do you work in/did you most recently work in? Examples are included. Please select the most appropriate category.**

**O** Hospital healthcare professional (physician, nurse, respiratory therapist, medical lab assistant, etc.)

**O** Hospital support staff (environment and cleaning, food service, etc.)

**O** Community healthcare professional (physical therapist, psychologist, dietician, etc.)

**O** Dental industry (dentist, hygienist, assistant, etc.)

**O** First responder (police, fire, paramedic services)

**O** Restaurant, bar, or nightclub industry

**O** Energy industry (oil, gas, solar, hydro, etc.)

**O** Service industry (grocery stores, hardware stores, liquor stores, etc.)

**O** Agricultural and natural resource sector (farming, logging, mining, etc.)

**O** Construction industry, other material building suppliers, and related trades (residential or

commercial, including a contractor or sub-contractor)

**O** Government / public service sector (federal, provincial, or municipal)

**O** Entertainment (music, arts, theatre, photography, etc.)

**O** Primary or secondary education (public or private)

**O** Post-secondary education, academia, and research (including support staff)

**O** Industry sponsor research (e.g. private companies)

**O** Other: **[PLEASE SPECIFY]** ________________

**O** Prefer not to answer

**D8. Has the government identified your occupation as an essential service?**

**O** Yes

**O** No

**O** Unsure

**O** Prefer not to answer

**D9. How many incomes contribute to your overall household income?**

**_____** income(s) [verification: integer]

**O** Prefer not to answer

**D10. What was your total overall household income in 2019 before taxes?**

**O** $0

**O** $1 to $9 999

**O** $10 000 to $24 999

**O** $25 000 to $49 999

**O** $50 000 to $74 999

**O** $75 000 to $99 999

**O** $100 000 to $149 999

**O** $150 000 to $249 999

**O** $250 000 or more

**O** Don’t know

**O** Prefer not to answer

**D11.** As you know, we all live in Canada, but we come from many different ethnic backgrounds. What is your **main** ethnic background? Please select up to two responses.

**O *Canadian/French Canadian***

**O *Caucasian/White***

**O *British*** (English/Scottish/Welsh/Irish)

**O *Western European*** (from Austria, Belgium, France, Germany, Netherlands, or other)

**O *Southern or Eastern European*** (from Greece, Italy, Portugal, Spain, Bosnia, Croatia, Serbia, Czech Republic, Hungary, Poland, Slovakia, Ukraine, former Soviet Republics, or other)

**O *South Asian*** (Punjabi, Indian, Tamil, Sri Lankan, Pakistani, Bangladeshi, Nepalese)

**O *East or Southeast Asian*** (from China, Hong Kong, Japan, North or South Korea, Indonesia, Malaysia, Philippines, Singapore, Thailand, Vietnam or other)

**O *West Asian or Middle Eastern*** (from Afghanistan, Iran, Iraq, Israel, Lebanon, Saudi Arabia, Syria, Turkey or other)

**O *African***

**O *Central/South American or Caribbean*** (from Argentina, Brazil, Columbia, El Salvador, Guatemala, Mexico, Venezuela, Barbados, Jamaica, or other)

**O *Aboriginal/First Nations/Métis***

**O** Other (Specify)

**O** Prefer not to answer

**D12.** Which of the following best describes your religious identity?

**[SINGLE PUNCH]**

**O** Roman Catholic

**O** Protestant or other Christian

**O** Muslim

**O** Jewish

**O** Hindu

**O** Sikh

**O** Other **[SPECIFY]**___________________

**O** No Religious Identity

**O** Don't know/Prefer not to answer

**D13. Thinking of how you feel right now, if a FEDERAL election were held tomorrow, which of the following parties' candidates would you, yourself, be most likely to support?***Please select one response only.*

**[RANDOMIZE CODES 1-5]**

**O** The Conservative Party
**O** The Liberal Party
**O** The New Democratic Party (NDP)
**O [QUÉBEC ONLY]** The Bloc Québécois (BQ)

**O** The Green Party
**O [SHOW FOURTH TO LAST]** Some other independent party

**O [SHOW THIRD TO LAST]** Would not vote/None/Would spoil ballot)
**O [SHOW SECOND TO LAST]** Don’t know/not sure

**O [SHOW LAST]** Prefer not to answer

**[IF DON'T KNOW OR NOT SURE IN QD13, ASK QD13b, ELSE SKIP TO QD14]**

**D13b. Well, which party would you say you would lean towards?** *Please select one response only.*

**[SAME ORDER AS QD13]**

**O** The Conservative Party
**O** The Liberal Party
**O** The New Democratic Party (NDP)
**O** The Green Party

**O [QUÉBEC ONLY]** The Bloc Québécois (BQ)
**O [SHOW THIRD TO LAST]** Some other independent party

**O [SHOW SECOND TO LAST]** Don’t know/not sure

**O [SHOW LAST]** Prefer not to answer

**D14. What is the highest level of education have you completed?**

**O** Less than a high school diploma

**O** High school diploma

**O** CEGEP

**O** Vocational college

**O** Trade certification

**O** Some college (no degree)

**O** College degree

**O** Some university (no degree)

**O** Undergraduate degree (Bachelor’s)

**O** Graduate degree (Masters or Doctorate)

**O** Professional degree (MD, JD, DDS, etc.)

**O** Prefer not to answer

**D15. What kind of residence do you live in?**

**O** Detached home

**O** Semi-detached home (e.g. duplex, townhouse)

**O** Apartment or condominium

**O** School residence or dormitories

**O** Assisted living or care home

**O** Other communal housing (e.g. transition house, shelter)

**O** Other: **[PLEASE SPECIFY]**__________

**O** Prefer not to answer

**[ASK QD16 IF QD15 = “Apartment or condominium”, “School residence or dormitories”, “Assisted living or care home”, or “Other communal housing (e.g. transition house, shelter”]**

**D16.** **Have any specific regulations or policies in response to COVID-19 been implemented in your building (e.g. supplemental cleaning procedures, elevator occupancy limits, etc.)?**

**O** Yes

**O** No

**O** Unsure

**O** Prefer not to answer

**D17. Are you currently diagnosed with any of the following health conditions? Please select all that apply.**

*Please do not select any conditions you may have been previously diagnosed but no longer have.*

**▢** Autoimmune disease (e.g. lupus, psoriasis, rheumatoid arthritis, Crohn’s disease, etc.)

**▢** Cancer (currently in treatment)

**▢** Chronic lung disease (e.g. COPD, asthma, etc.)

**▢** Dementia

**▢** Diabetes (Type 1 or Type 2)

**▢** Cardiovascular disease (e.g. arrhythmias, coronary artery diseases, hypertension, valvular diseases, etc., including congenital diseases)

**▢** Mental health (e.g. anxiety, depression, post-traumatic stress disorder, substance use disorder, etc.)

**▢** Obesity

**▢** Other chronic diseases (e.g. high cholesterol, kidney (renal) disease, osteoarthritis, neuromuscular, etc.)

**▢** Other: **[PLEASE SPECIFY]**__________

**O** I am not currently diagnosed with any of these health conditions

**O** Prefer not to answer

**Thank you for your participation.**

**Your participation provides important insights that will be used to help improve the COVID-19 pandemic response. For more information about COVID-19 please visit please visit:** [**www.Canada.ca/covid-19**](http://www.Canada.ca/covid-19) **.**

**If you have questions, please contact** [**C3ResNetwork@ucalgary.ca**](mailto:c3ResNetwork@ucalgary.ca)**.**

**SUBMIT**

**QUESTIONNAIRE PRINCIPAL**

1. Êtes-vous au courant de la pandémie de COVID-19?

- Oui
- Non

[IF Q.1=“NO”, GO TO ‘READ SCREENa’ AT THE BEGINNING OF THE DEMOGRAPHIC SECTION]

Nous aimerions vous poser des questions sur le nouveau coronavirus qui cause la COVID-19. Ce virus est parfois appelé SRAS-CoV-2 (coronavirus du syndrome respiratoire aigu sévère 2).

Nous aimerions connaître vos points de vue, votre expérience et votre compréhension de la COVID-19.

Veuillez répondre à toutes les questions sans consulter Internet ni aucune autre source.

2. Êtes-vous ou avez-vous été malade et pensé ou avez pensé être atteint de la COVID-19 (en ce moment ou antérieurement)?

- Oui, et j’ai reçu un résultat de test de dépistage positif
- Oui, mais je n’ai pas subi de test de dépistage
- Oui, mais j’ai reçu un résultat de test de dépistage négatif
- Non
- Je ne sais pas
- Je préfère ne pas répondre

3. Connaissez-vous quelqu’un qui a reçu un résultat de test de dépistage positif pour la COVID-19? Veuillez sélectionner toutes les réponses qui s’appliquent.

- Oui, des membres de la famille qui vivent avec moi
- Oui, des membres de la famille qui ne vivent pas avec moi
- Oui, des amis proches
- Oui, des collègues de travail ou des connaissances
- Non, je ne connais personnellement personne qui a reçu un diagnostic de COVID-19
- Je préfère ne pas répondre

4. Dans quelle mesure la COVID-19 représente-t-elle un problème grave au Canada en ce moment?

| O | O | O | O | O | O |
| --- | --- | --- | --- | --- | --- |
| Pas grave | Un peu grave | Plutôt grave | Modérément grave | Très grave | Je préfère ne pas répondre |

5. Dans quelle mesure la COVID-19 représente-t-elle un problème grave au Canada comparativement à dans d’autres pays du monde?

| O | O | O | O | O | O |
| --- | --- | --- | --- | --- | --- |
| Beaucoup moins grave | Un peu moins grave | À peu près aussi grave | Un peu plus grave | Beaucoup plus grave | Je préfère ne pas répondre |

6. Au début de l’année 2020, comment auriez-vous évalué les aspects suivants de votre santé générale :

[RANDOMIZE ITEMS]

|  | Mauvaise | Assez bonne | Bonne | Très bonne | Excellente | Je préfère ne pas répondre |
| --- | --- | --- | --- | --- | --- | --- |
| Santé mentale/émotionnelle | O | O | O | O | O | O |
| Santé physique | O | O | O | O | O | O |
| Santé sociale | O | O | O | O | O | O |
| Santé économique | O | O | O | O | O | O |
| Santé spirituelle | O | O | O | O | O | O |

7. Au présent, comment évalueriez-vous les aspects suivants de votre santé générale :

[ASK ITEMS IN Q.7 IN THE SAME ORDER AS RANDOMIZED IN Q.6]

|  | Mauvaise | Assez bonne | Bonne | Très bonne | Excellente | Je préfère ne pas répondre |
| --- | --- | --- | --- | --- | --- | --- |
| Santé mentale/émotionnelle | O | O | O | O | O | O |
| Santé physique | O | O | O | O | O | O |
| Santé sociale | O | O | O | O | O | O |
| Santé économique | O | O | O | O | O | O |
| Santé spirituelle | O | O | O | O | O | O |

8. Veuillez indiquer dans quelle mesure vous êtes d’accord avec chacun des énoncés suivants.

[RANDOMIZE ITEMS 8.1, 8.2 AND 8.3. ITEMS 8.4 AND 8.5 ARE ALWAYS ASKED LAST]

|  | Tout à fait en désaccord | En désaccord | Plutôt en désaccord | Je suis indécis(e) | Plutôt d’accord | D’accord | Tout à fait d’accord | Je préfère ne pas répondre/Je ne sais pas |
| --- | --- | --- | --- | --- | --- | --- | --- | --- |
| La pandémie de COVID-19 m’inspire un sentiment d’impuissance | O | O | O | O | O | O | O | O |
| La pandémie de COVID-19 est très stressante | O | O | O | O | O | O | O | O |
| Le gouvernement fédéral n’en fait PAS assez pour soutenir les personnes touchées par la COVID-19 | O | O | O | O | O | O | O | O |
| Mon gouvernement provincial n’en fait PAS assez pour soutenir les personnes touchées par la | O | O | O | O | O | O | O | O |

9. Veuillez indiquer dans quelle mesure chacun des énoncés suivants vous cause de l’inquiétude.

[ASK 9.1 ONLY IF Q.2=“Yes, but I tested negative”, “No” or “Don’t know”]

[ALWAYS ASK 9.1 AND 9.2 FIRST]

[RANDOMIZE ITEMS 9.3, 9.4 AND 9.5]

|  | Pas du tout | Un petit peu | Modérément | Très bonne | Énormément | Je préfère ne pas répondre/Sans objet |
| --- | --- | --- | --- | --- | --- | --- |
| Je vais contracter la COVID-19 | O | O | O | O | O | O |
| Un membre de ma famille va contracter la COVID-19 | O | O | O | O | O | O |
| Les gens ne pourront pas avoir accès aux soins de santé dont ils ont besoin en raison des restrictions associées à la COVID-19 | O | O | O | O | O | O |
| Il n’y aura pas suffisamment d’équipement de protection (comme des masques) pour assurer la sécurité du personnel hospitalier | O | O | O | O | O | O |
| Il n’y aura pas suffisamment d’équipement hospitalier (comme des lits) pour prendre soin des patients atteints de la COVID-19 | O | O | O | O | O | O |

10. Qu’est-ce qui vous inquiète le plus par rapport à la pandémie de COVID-19?

[Open ended response to be coded]

- Je préfère ne pas répondre

11. Comment évalueriez-vous votre compréhension générale de ce qui cause la propagation du virus à l’origine de la COVID-19?

| O | O | O | O | O | O |
| --- | --- | --- | --- | --- | --- |
| Mauvaise | Assez bonne | Bonne | Très bonne | Excellente | Je préfère ne pas répondre |

12. Veuillez indiquer dans quelle mesure vous êtes d’accord avec chacun des énoncés suivants sur la transmission du virus à l’origine de la COVID-19 :

[RANDOMIZE ITEMS]

|  | Tout à fait en désaccord | En désaccord | Plutôt en désaccord | Je suis indécis(e) | Plutôt d’accord | D’accord | Tout à fait d’accord | Je préfère ne pas répondre/Je ne sais pas |
| --- | --- | --- | --- | --- | --- | --- | --- | --- |
| Le virus peut se propager par l’intermédiaire des aliments et des boissons | O | O | O | O | O | O | O | O |
| Il est possible d’être infecté par le virus de la COVID-19 sans présenter aucun symptôme | O | O | O | O | O | O | O | O |
| Le virus se propage uniquement en cas de contact étroit avec une personne qui présente des symptômes de COVID-19 | O | O | O | O | O | O | O | O |
| Le port de gants en public peut contribuer à prévenir la propagation du virus | O | O | O | O | O | O | O | O |
| Nous ne pouvons rien faire pour arrêter la propagation du virus; nous finirons tous par contracter la COVID-19 | O | O | O | O | O | O | O | O |
| Le protocole de dépistage de la COVID-19 (personnes qui reçoivent les tests, nombre de tests) dans ma province est suffisant | O | O | O | O | O | O | O | O |

13. À quelle fréquence cherchez-vous à vous renseigner sur la COVID-19?

| O | O | O | O | O | O |
| --- | --- | --- | --- | --- | --- |
| Jamais | Une fois par semaine | Tous les deux ou trois jours | Une fois par jour | Plusieurs fois par jour | Je préfère ne pas répondre |

14. Quelles sources avez-vous consultées pour obtenir des renseignements sur la COVID-19 au cours des deux dernières semaines? Veuillez sélectionner toutes les sources que vous avez consultées.

Sites Web du gouvernement et de la santé publique

▢ Sites Web nationaux canadiens

▢ Sites Web provinciaux canadiens

▢ Sites Web gouvernementaux américains

▢ Site Web de l’Organisation mondiale de la santé

Télévision, médias imprimés ou sites Web

▢ Nouvelles télévisées d’un réseau canadien

▢ Nouvelles télévisées d’un réseau américain

▢ Magazines/journaux canadiens

▢ Magazines/journaux américains

▢ Sites Web de nouvelles canadiens

▢ Sites Web de nouvelles américains

▢ Sources de nouvelles internationales, y compris des sites Web, des émissions et des journaux

▢ Autres sites Web

Médias sociaux

▢ Publications d’organismes de santé et du gouvernement dans les médias sociaux

▢ Publications d’amis et de membres de la famille dans les médias sociaux

▢ Publications d’influenceurs ou de célébrités dans les médias sociaux

Bouche-à-oreille

▢ Conversations avec des amis ou des membres de la famille

▢ Conversations avec des collègues de travail

▢ Conversations avec des connaissances ou des personnes qui ne font pas partie de mon entourage

▢ Conversations avec un professionnel de la santé

▢ Autre :_________________

- - Je n’ai consulté aucune source d’information sur la COVID-19 au cours des deux dernières semaines
  - Aucune de ces réponses
  - Je préfère ne pas répondre

15. Quels types de renseignements avez-vous recherchés au sujet de la pandémie de COVID-19 au cours des deux dernières semaines? Veuillez sélectionner toutes les réponses qui s’appliquent.

▢ Symptômes de la COVID-19

▢ Taux d’infection

▢ Taux et protocole de dépistage

▢ Témoignages de personnes sur la COVID-19

▢ Témoignages de personnes qui sont ou ont été atteintes de la COVID-19

▢ Avancée de la mise au point d’un vaccin contre la COVID-19 et innocuité d’un vaccin potentiel

▢ Traitements actuellement utilisés ou en cours de mise au point contre la COVID-19

▢ Façons dont je peux personnellement prévenir la propagation de la maladie

▢ Accès aux services sociaux ou aux ressources

▢ Façons de prendre soin d’une personne à risque

▢ Renseignements sur l’éducation de mes enfants

▢ Restrictions liées aux voyages

▢ Autre : _____________

O Je n’ai recherché aucun renseignement sur la COVID-19 au cours des deux dernières semaines

O Aucun de ces sujets

O Je préfère ne pas répondre

[PN: PLEASE USE DRAG AND DROP FOR Q.18]

16. Veuillez sélectionner et classer selon le degré de fiabilité, les trois sources LES PLUS fiables et les trois sources LES MOINS fiables pour obtenir des renseignements sur la COVID-19.

Sites Web du gouvernement et de la santé publique

▢ Sites Web nationaux canadiens

▢ Sites Web provinciaux canadiens

▢ Sites Web gouvernementaux américains

▢ Site Web de l’Organisation mondiale de la santé

Télévision, médias imprimés ou sites Web

▢ Nouvelles télévisées d’un réseau canadien

▢ Nouvelles télévisées d’un réseau américain

▢ Magazines/journaux canadiens

▢ Magazines/journaux américains

▢ Sites Web de nouvelles canadiens

▢ Sites Web de nouvelles américains

▢ Sources de nouvelles internationales, y compris des sites Web, des émissions et des journaux

▢ Autres sites Web

Médias sociaux

▢ Publications d’organismes de santé et du gouvernement dans les médias sociaux

▢ Publications d’amis et de membres de la famille dans les médias sociaux

▢ Publications d’influenceurs ou de célébrités dans les médias sociaux

Bouche-à-oreille

▢ Conversations avec des amis ou des membres de la famille

▢ Conversations avec des collègues de travail

▢ Conversations avec des connaissances ou des personnes qui ne font pas partie de mon entourage

▢ Conversations avec un professionnel de la santé

▢ Autre : __________

O Il n’y a pas de sources fiables

O Aucune de ces sources

O Je préfère ne pas répondre

17. Dans quelle mesure croyez-vous être capable de repérer les renseignements inexacts ou trompeurs sur la COVID-19?

| O | O | O | O | O | O |
| --- | --- | --- | --- | --- | --- |
| Pas du tout | Un peu | Assez | Modérément | Extrêmement | Je préfère ne pas répondre |

18. Dans quelle mesure croyez-vous qu’une personne moyenne est capable de repérer les renseignements inexacts ou trompeurs sur la COVID-19?

| O | O | O | O | O | O |
| --- | --- | --- | --- | --- | --- |
| Pas du tout | Un peu | Assez | Modérément | Extrêmement | Je préfère ne pas répondre |

19. Avez-vous vu ou entendu des renseignements inexacts ou trompeurs sur la COVID-19 au cours des deux dernières semaines? Veuillez sélectionner tous les types de renseignements qui s’appliquent.

▢ Symptômes de la COVID-19

▢ Taux d’infection

▢ Taux et protocole de dépistage

▢ Témoignages de personnes sur la COVID-19

▢ Témoignages de personnes qui sont ou ont été atteintes de la COVID-19

▢ Avancée de la mise au point d’un vaccin contre la COVID-19 et innocuité d’un vaccin potentiel

▢ Traitements actuellement utilisés ou en cours de mise au point contre la COVID-19

▢ Façons dont je peux personnellement prévenir la propagation de la maladie

▢ Accès aux services sociaux ou aux ressources

▢ Façons de prendre soin d’une personne à risque

▢ Renseignements sur l’éducation de mes enfants

▢ Restrictions liées aux voyages

▢ Autre : _________________________

O Je n’ai pas vu ni entendu de renseignements inexacts ou trompeurs liés à la COVID-19 au cours des deux dernières semaines

O Je préfère ne pas répondre

[ASK Q.21.1 IF Q21 IS NOT “I did not see or hear any incorrect or misleading information related to COVID-19 the last 2 weeks” or “Prefer not to answer”]

20. Où avez-vous vu ou entendu des renseignements inexacts ou trompeurs? Veuillez sélectionner toutes les réponses qui s’appliquent.

Sites Web du gouvernement et de la santé publique

▢ Sites Web nationaux canadiens

▢ Sites Web provinciaux canadiens

▢ Sites Web gouvernementaux américains

▢ Site Web de l’Organisation mondiale de la santé

Télévision, médias imprimés ou sites Web

▢ Nouvelles télévisées d’un réseau canadien

▢ Nouvelles télévisées d’un réseau américain

▢ Magazines/journaux canadiens

▢ Magazines/journaux américains

▢ Sites Web de nouvelles canadiens

▢ Sites Web de nouvelles américains

▢ Sources de nouvelles internationales, y compris des sites Web, des émissions et des journaux

▢ Autres sites Web

Médias sociaux

▢ Publications d’organismes de santé et du gouvernement dans les médias sociaux

▢ Publications d’amis et de membres de la famille dans les médias sociaux

▢ Publications d’influenceurs ou de célébrités dans les médias sociaux

Bouche-à-oreille

▢ Conversations avec des amis ou des membres de la famille

▢ Conversations avec des collègues de travail

▢ Conversations avec des connaissances ou des personnes qui ne font pas partie de mon entourage

▢ Conversations avec un professionnel de la santé

▢ Autre : __________

O Aucune de ces sources

O Je ne m’en souviens pas

O Je préfère ne pas répondre

22. Comment vérifiez-vous ou validez-vous les renseignements que vous voyez ou entendez? Veuillez sélectionner toutes les réponses qui s’appliquent.

▢ J’utilise un moteur de recherche en ligne (p. ex., Google, Yahoo)

▢ Je consulte directement une source de nouvelles en ligne

▢ Je consulte directement une source du gouvernement ou des autorités sanitaires

▢ Je consulte des articles scientifiques

▢ Je demande à un membre de la famille ou à un ami

▢ Je demande à un professionnel de la santé

▢ Je pose des questions sur la crédibilité des renseignements dans les médias sociaux

▢ J’utilise Snopes ou un autre service de vérification des faits

▢ Je n’utilise aucune des stratégies ci-dessus

▢ Autre : __________

O Je ne vérifie pas les renseignements, car je choisis simplement de ne pas les croire

O Je ne vérifie pas les renseignements, car je choisis simplement de les croire

O Je préfère ne pas répondre

23. Veuillez indiquer dans quelle mesure vous êtes d’accord avec chacun des énoncés suivants.

[RANDOMIZE ITEMS]

|  | Tout à fait en désaccord | En désaccord | Plutôt en désaccord | Je suis indécis(e) | Plutôt d’accord | D’accord | Tout à fait d’accord | Je préfère ne pas répondre/Je ne sais pas |
| --- | --- | --- | --- | --- | --- | --- | --- | --- |
| Je suis en mesure de trouver les renseignements que je veux au sujet de la COVID-19 | O | O | O | O | O | O | O | O |
| Je trouve qu’il est difficile de déterminer si une source d’information est fiable ou non |  |  |  |  |  |  |  |  |

24. Êtes-vous en isolement volontaire en ce moment?

*L’isolement volontaire consiste à vous isoler des autres, y compris des membres de votre foyer, dans le but de prévenir la propagation du virus (que vous ayez ou non reçu un diagnostic ou présentiez ou non des symptômes). Vous ne quittez pas la maison, ne vous rendez pas au travail, n’allez pas à l’épicerie, ni n’accédez à d’autres services essentiels.*

- - Oui, je suis en isolement volontaire en ce moment
  - Non, je ne suis pas en isolement volontaire en ce moment
  - Je préfère ne pas répondre

[ASK Q.25 IF Q.24 = “No, I am not currently in self-isolation” or “Prefer not to answer”]

25. À quelle fréquence respectez-vous la distanciation sociale ou physique?

*La distanciation sociale ou physique consiste à limiter le temps que vous passez dans des espaces occupés par d’autres personnes, y compris la réduction de vos déplacements pour rendre visite à d’autres personnes et du temps que vous passez dans les lieux publics.*

*Selon les recommandations de l’OMS, « distanciation sociale » serait le terme le plus approprié à utiliser pour décrire ces mesures sociales. Par conséquent, nous utiliserons le terme « distanciation sociale » pour le reste du sondage.*

| O | O | O | O | O | O |
| --- | --- | --- | --- | --- | --- |
| Jamais | Rarement | Parfois | Souvent | Toujours | Je préfère ne pas répondre |

[ASK Q.26 IF Q.24 = “Yes, I am currently in self-isolation”]

26. Pourquoi êtes-vous en isolement volontaire? Veuillez sélectionner jusqu’à trois raisons principales.

▢ Pour me protéger

▢ Pour protéger les personnes avec qui je vis

▢ Pour protéger les autres personnes avec qui je vis qui sont vulnérables/à risque élevé

▢ Pour protéger d’autres membres de ma famille ou amis qui ne vivent pas avec moi

▢ Pour protéger la population générale

▢ Pour contribuer à réduire le fardeau sur le réseau de la santé

▢ Pour éviter de m’exposer à une amende pour ne pas avoir suivi les directives

▢ Autre : __________

O Je préfère ne pas répondre

[ASK Q.27 IF Q.25 = “Sometimes”, “Often” or “Always”]

27. Pourquoi respectez-vous la distanciation sociale? Veuillez sélectionner jusqu’à trois raisons principales.

▢ Pour me protéger

▢ Pour protéger les personnes avec qui je vis

▢ Pour protéger les autres personnes avec qui je vis qui sont vulnérables/à risque élevé

▢ Pour protéger d’autres membres de ma famille ou amis qui ne vivent pas avec moi

▢ Pour protéger la population générale

▢ Pour contribuer à réduire le fardeau sur le réseau de la santé

▢ Pour éviter de m’exposer à une amende pour ne pas avoir suivi les directives

▢ Autre : __________

O Je préfère ne pas répondre

[ASK Q.28 IF Q.25 = “Not at all” or “Rarely”]

28. Pourquoi ne respectez-vous pas régulièrement la distanciation sociale? Veuillez sélectionner toutes les réponses qui s’appliquent.

▢ Je suis en santé

▢ Je ne crains pas de contracter le virus à l’origine de la COVID-19

▢ Je crois que la situation n’est pas aussi terrible que les gens l’affirment

▢ Il est important pour moi de continuer de rendre visite à mes amis ou aux membres de ma famille pendant la pandémie

▢ Mon travail fait en sorte qu’il est très difficile de respecter la distanciation sociale

▢ Ce n’est pas important puisque toute la population finira par contracter le virus à l’origine de la COVID-19

▢ Autre : __________

O Je préfère ne pas répondre

[ASK Q.29 IF Q.26 = “To protect other people that I live with who are vulnerable” OR IF Q.27 = “To protect other people that I live with who are vulnerable”]

29. Vous avez indiqué que vous êtes en isolement volontaire ou que vous respectez la distanciation sociale ou physique pour protéger une personne vulnérable ou à risque élevé. Veuillez décrire les personnes vulnérables que vous protégez en étant en isolement volontaire ou en respectant la distanciation sociale ou physique et expliquer pourquoi elles sont vulnérables.

[Open ended response to be coded]

O Je préfère ne pas répondre

[If Q.24 = “Yes, I am currently in self-isolation”, include READ SCREEN below]

[READ SCREEN]

*La distanciation sociale ou physique consiste à limiter le temps que vous passez dans des espaces occupés par d’autres personnes, y compris la réduction de vos déplacements pour rendre visite à d’autres personnes et du temps que vous passez dans les lieux publics. Selon les recommandations de l’OMS, « distanciation sociale » serait le terme le plus approprié à utiliser pour décrire ces mesures sociales. Par conséquent, nous utiliserons le terme « distanciation sociale » pour le reste du sondage.*

[ASK ALL]

30. À votre avis, quel est le groupe d’âge qui respecte la distanciation sociale avec le plus de constance?

O Adolescents

O Jeunes adultes

O Adultes d’âge moyen

O Aînés

O Je ne sais pas

O Je préfère ne pas répondre

31. À votre avis, quel est le groupe d’âge qui respecte la distanciation sociale avec le moins de constance?

O Adolescents

O Jeunes adultes

O Adultes d’âge moyen

O Aînés

O Je ne sais pas

O Je préfère ne pas répondre

32. Pendant encore combien de temps estimez-vous que vous pourrez raisonnablement maintenir votre niveau de distanciation sociale actuel?

O Je ne peux pas le maintenir plus longtemps

O Encore deux semaines

O Encore un mois

O Encore deux mois

O Encore trois mois

O Encore six mois

O Encore plus de six mois (aussi longtemps que ce sera nécessaire)

O Je ne sais pas

O Je préfère ne pas répondre

33. Veuillez indiquer dans quelle mesure vous êtes d’accord avec chacun des énoncés suivants.

[RANDOMIZE ITEMS]

|  | Tout à fait en désaccord | En désaccord | Plutôt en désaccord | Je suis indécis(e) | Plutôt d’accord | D’accord | Tout à fait d’accord | Je préfère ne pas répondre/Je ne sais pas |
| --- | --- | --- | --- | --- | --- | --- | --- | --- |
| En modifiant mon comportement, je contribue efficacement à prévenir la propagation du virus à l’origine de la COVID-19 | O | O | O | O | O | O | O | O |
| Je réussis mieux que la plupart des Canadiens à modifier mon comportement pour prévenir la propagation du virus à l’origine de la COVID-19 | O | O | O | O | O | O | O | O |
| Je vais me faire vacciner contre le virus lorsqu’un vaccin sera mis au point | O | O | O | O | O | O | O | O |

*Vous avez atteint la fin de cette partie du sondage. Merci pour vos réponses!*

Données démographiques

[READ SCREENa IF Q1=NO]

*Une pandémie de maladie respiratoire causée par le nouveau coronavirus, également appelée la COVID-19, sévit en ce moment. Le virus a été découvert pour la première fois à Wuhan, dans la province du Hubei, en Chine. Depuis, il s’est répandu partout dans le monde. On dénombre des centaines de milliers de cas confirmés et de nombreux décès liés à la COVID-19, y compris au Canada. Veuillez visiter le site Web du gouvernement du Canada sur la COVID-19 pour obtenir des renseignements.*

[READ SCREEN FOR ALL]

Nous aimerions maintenant vous poser des questions démographiques pour faciliter le regroupement des réponses. Nous vous rappelons que vos réponses seront utilisées uniquement dans le cadre d’une analyse regroupée des données. Nous ne serons pas en mesure de vous identifier.

D1. Quel est votre état matrimonial actuel?

O Célibataire/jamais marié(e)

O En couple, mais pas en union de fait

O En union de fait

O Marié(e)

O Séparé(e) ou divorcé(e) (processus en cours ou officialisé)

O Veuf/Veuve

O Je préfère ne pas répondre

D2. Combien d’habitants compte la ville où vous habitez?

O Région indépendante (p. ex., canton ou village – jusqu’à 1 000 habitants)

O Petite municipalité ou village (jusqu’à 5 000 habitants)

O Petite ville (jusqu’à 10 000 habitants)

O Ville moyenne (de plus de 10 000 habitants à 100 000 habitants)

O Grande ville (de plus de 100 000 habitants à 1 000 000 d’habitants)

O Grande région métropolitaine (plus de 1 000 000 d’habitants)

O Je ne sais pas

O Je préfère ne pas répondre

D3. Vous compris, combien de personnes compte votre foyer? (Veuillez tenir compte de tous les autres membres de la famille ou colocataires vivant sous le même toit)

___ personne(s) [verification: integer]

O Je préfère ne pas répondre

D4. Combien d’enfants (âgés de moins de 18 ans) vivent dans votre foyer? Veuillez ne pas tenir compte des enfants qui étudient à l’extérieur. (Si vous n’avez pas d’enfants appartenant à un groupe d’âge en particulier, veuillez inscrire « 0 ».)

O Je n’ai pas d’enfants âgés de moins de 18 ans qui vivent dans mon foyer

___ nourrisson(s) (1 an ou moins) [verification: integer]

___ tout-petit(s) (1 ou 2 ans) [verification: integer]

___ enfant(s) (de 3 à 12 ans) [verification: integer]

___ adolescent(s) (de 13 à 17 ans) [verification: integer]

O Je préfère ne pas répondre

D5. Quelle est votre situation professionnelle actuelle? Veuillez sélectionner toutes les réponses qui s’appliquent.

▢ Employé(e) (à temps plein)

▢ Employé(e) (à temps partiel/occasionnel)

▢ Travailleur/travailleuse autonome (à temps plein)

▢ Travailleur/travailleuse autonome (à temps partiel)

▢ Retraité(e)

▢ Étudiant(e) (à temps plein ou à temps partiel)

▢ Parent ou personne au foyer à temps plein

▢ Militaire (à temps plein ou à temps partiel)

▢ Actuellement sans emploi ou dans l’incapacité de travailler pour quelque raison que ce soit (y compris une mise à pied)

O Je préfère ne pas répondre

[ASK QD6 IF D5 = “Currently unemployed or unable to work for any reason (including laid off)”]

D6. Le fait que vous soyez actuellement sans emploi représente-t-il une conséquence directe de la pandémie de COVID-19 (p. ex., mise à pied d’une entreprise, cessation des activités demandée par le gouvernement)?

O Oui

O Non

O Je ne suis pas certain(e)

O Je préfère ne pas répondre

[ASK D7 IF D5 = “Employed (working full-time hours)”, “Employed (working part-time/casual hours”, “Self-employed (working full-time hours)”, “Self-employed (working part-time hours)”, “Military (full or part-time)” or “Currently unemployed or unable to work for any reason (including laid off)”]

D7. Dans quel type de secteur travaillez-vous/travailliez-vous le plus récemment? Voici des exemples. Veuillez sélectionner la catégorie la plus pertinente.

O Professionnel(le) de la santé en milieu hospitalier (médecin, infirmier/infirmière, inhalothérapeute, technicien de laboratoire médical, etc.)

O Personnel de soutien en milieu hospitalier (environnement et entretien, service alimentaire, etc.)

O Professionnel(le) de la santé dans la communauté (ergothérapeute, psychologue, diététiste, etc.)

O Secteur dentaire (dentiste, hygiéniste, assistant(e), etc.)

O Premier répondant/première répondante (service de police, service des incendies, ambulancier/ambulancière)

O Secteur des restaurants, bars et boîtes de nuit

O Secteur de l’énergie (pétrole, gaz, énergie solaire, hydroélectricité, etc.)

O Secteur des services (épiceries, quincailleries, magasins vendant de l’alcool, etc.)

O Secteur de l’agriculture et des ressources naturelles (agriculture, exploitation forestière, mines, etc.)

O Secteur de la construction, autres fournisseurs importants en construction et métiers connexes (secteur résidentiel ou commercial, y compris les entrepreneurs et les sous-traitants)

O Gouvernement/secteur de la fonction publique (échelle fédérale, provinciale ou municipale)

O Divertissement (musique, arts, théâtre, photographie, etc.)

O Écoles primaires ou secondaires (publiques ou privées)

O Établissements d’éducation postsecondaires, universités et établissements de recherche (y compris le personnel de soutien)

O Recherche de commanditaires sectoriels (p. ex., entreprises privées)

O Autre : ________________

O Je préfère ne pas répondre

D8. Votre emploi figure-t-il dans la liste des services essentiels établie par le gouvernement?

O Oui

O Non

O Je ne suis pas certain(e)

O Je préfère ne pas répondre

D9. Combien de sources de revenus contribuent au revenu total de votre foyer?

_____ sources de revenus [verification: integer]

O Je préfère ne pas répondre

D10. Quel a été le revenu total de votre foyer en 2019, avant impôts?

O 0 $

O De 1 $ à 9 999 $

O De 10 000 $ à 24 999 $

O De 25 000 $ à 49 999 $

O De 50 000 $ à 74 999 $

O De 75 000 $ à 99 999 $

O De 100 000 $ à 149 999 $

O De 150 000 $ à 249 999 $

O 250 000 $ ou plus

O Je ne sais pas

O Je préfère ne pas répondre

D11. Comme vous le savez, nous vivons tous au Canada, mais nous appartenons à des groupes ethniques différents. Quelle est votre origine ethnique principale? Veuillez sélectionner deux réponses au maximum.

O Canadienne/Canadienne française

O Caucasienne/blanche

O Britannique (Angleterre, Écosse, Pays de Galles, Irlande)

O Européenne occidentale (Autriche, Belgique, France, Allemagne, Pays-Bas ou autre)

O Européenne australe ou orientale (Grèce, Italie, Portugal, Espagne, Bosnie, Croatie, Serbie, République tchèque, Hongrie, Pologne, Slovaquie, Ukraine, anciennes républiques soviétiques ou autre)

O Asiatique australe (Punjab, Inde ou Sri Lanka [tamoul], Pakistan, Bangladesh, Népal)

O Extrême-orientale ou asiatique du Sud-Est (Chine, Hong Kong, Japon, Corée du Nord ou du Sud, Indonésie, Malaisie, Philippines, Singapour, Thaïlande, Vietnam ou autre)

O Asiatique occidentale ou moyen-orientale (Afghanistan, Iran, Iraq, Israël, Liban, Arabie Saoudite, Syrie, Turquie ou autre)

O Africaine

O Centraméricaine, sud-américaine ou caribéenne (Argentine, Brésil, Colombie, El Salvador, Guatemala, Mexique, Venezuela, Barbade, Jamaïque ou autre)

O Autochtone/membre des Premières Nations/Métis

O Autre (veuillez préciser)

O Je préfère ne pas répondre

D12. Parmi les religions suivantes, laquelle décrit le mieux votre identité religieuse?

[SINGLE PUNCH]

O Catholique

O Protestante ou autre religion chrétienne

O Musulmane

O Juive

O Hindoue

O Sikhe

O Autre [SPECIFY]___________________

O Je ne m’identifie à aucune religion

O Je ne sais pas/Je préfère ne pas répondre

D13. En pensant à ce que vous ressentez maintenant, si une élection FÉDÉRALE était tenue demain, de quel parti seriez-vous le plus susceptible d’appuyer le candidat? Veuillez choisir une seule réponse.

[RANDOMIZE CODES 1-5]

O Le Parti conservateur

O Le Parti libéral

O Le Nouveau Parti démocratique (NPD)

O [QUÉBEC ONLY] Le Bloc québécois (BQ)

O Le Parti vert

O [SHOW FOURTH TO LAST] Un autre parti indépendant

O [SHOW THIRD TO LAST] Je ne voterais pas/aucun/j’annulerais mon vote

O [SHOW SECOND TO LAST] Je ne sais pas/Je ne suis pas certain(e)

O [SHOW LAST] Je préfère ne pas répondre

[IF DON’T KNOW OR NOT SURE IN QD13, ASK QD13b, ELSE SKIP TO QD14]

D13b. Et pour quel parti diriez-vous que vous auriez tendance à voter? Veuillez sélectionner une seule réponse.

[SAME ORDER AS QD13]

O Le Parti conservateur

O Le Parti libéral

O Le Nouveau Parti démocratique (NPD)

O Le Parti vert

O [QUÉBEC ONLY] Le Bloc québécois (BQ)

O [SHOW THIRD TO LAST] Un autre parti indépendant

O [SHOW SECOND TO LAST] Je ne sais pas/Je ne suis pas certain(e)

O [SHOW LAST] Je préfère ne pas répondre

D14. Quel est le plus haut niveau d’études que vous avez atteint?

O Études secondaires partielles ou moins

O Diplôme d’études secondaires

O CÉGEP

O École professionnelle

O Certificat d’une école de métiers

O Études collégiales partielles (aucun diplôme)

O Diplôme d’études collégiales

O Études universitaires partielles (aucun diplôme)

O Diplôme d’études universitaires de premier cycle (baccalauréat)

O Diplôme d’études supérieures (maîtrise ou doctorat)

O Diplôme professionnel (M.D, D.D.S, J.D., etc.)

O Je préfère ne pas répondre

D15. Dans quel type de résidence habitez-vous?

O Maison isolée

O Maison jumelée (p. ex., duplex, maison en rangée)

O Appartement ou appartement en copropriété

O Résidence ou dortoir en milieu scolaire

O Résidence-services ou établissement de soins

O Autre logement communautaire (p. ex., maison de transition, refuge)

O Autre : __________

O Je préfère ne pas répondre

[ASK QD16 IF QD15 = “Apartment or condominium”, “School residence or dormitories”, “Assisted living or care home”, or “Other communal housing (e.g. transition house, shelter”]

D16. Des règles ou politiques particulières ont-elles été déployées dans votre immeuble en réponse à la pandémie de COVID-19 (p. ex., procédures de nettoyage supplémentaires, limites de personnes dans les ascenseurs)?

O Oui

O Non

O Je ne suis pas certain(e)

O Je préfère ne pas répondre

D17. Avez-vous un diagnostic de l’un ou l’autre des problèmes de santé suivants à l’heure actuelle? Veuillez sélectionner toutes les réponses qui s’appliquent.

Veuillez ne pas sélectionner de problèmes pour lesquels vous avez reçu un diagnostic antérieur, mais dont vous n’êtes plus atteint(e).

▢ Maladie auto-immune (p. ex., lupus, psoriasis, polyarthrite rhumatoïde, maladie de Crohn)

▢ Cancer (traitement en cours)

▢ Maladie pulmonaire chronique (p. ex., MPOC, asthme)

▢ Démence

▢ Diabète (type 1 ou 2)

▢ Maladie cardiovasculaire (p. ex., arythmie, coronaropathie, hypertension, valvulopathie, y compris les maladies congénitales)

▢ Trouble de santé mentale (p. ex., anxiété, dépression, trouble de stress post-traumatique, alcoolisme, toxicomanie)

▢ Obésité

▢ Autres maladies chroniques (p. ex., hypercholestérolémie, néphropathie [rein], arthrose, maladie neuromusculaire)

▢ Autre : __________

O Je n’ai pas de diagnostic de l’un ou l’autre de ces problèmes de santé à l’heure actuelle

O Je préfère ne pas répondre

SOUMETTRE
