# Supplementary material for: A national cross-sectional survey of public perceptions of the COVID-19 pandemic: Self-reported beliefs, knowledge, and behaviors
Source: PLoS One. 2020 Oct 23;15(10):e0241259. doi: 10.1371/journal.pone.0241259 (PMC7584165; doi:10.1371/journal.pone.0241259)
Supplement: S2 Appendix — (DOCX) [file pone.0241259.s002.docx]

**S2 Appendix Additional Aggregate Data Figures and Tables**

Table A. Self-reported perceptions, knowledge, and behaviors [2](#TableS2)

Figure A. Perceived psychological impact of COVID-19 and sufficiency of government response [3](#FigureA)

Figure B. Proportion of respondents who used various strategies to fact check misinformation [4](#FigureB)

Figure C. Respondents’ evaluation of information seeking and preventative behaviors [5](#FigureC)

Figure D. Topics searched for and topics identified as misinformation [6](#FigureD)

Figure E. Confidence in self and others ability to identify misinformation [7](#FigureE)

Figure F. Perceptions of age groups most and least consistently practicing physical distancing [8](#FigureF)

**Table A:** Self-reported perceptions, knowledge, and behaviors (total sample size=1,996)

| **Question** | **Weighted Frequency (%)^[[1]](#footnote-1)^** |
| --- | --- |
| **Perceptions of COVID-19** |  |
| How serious of a problem is COVID-19 in Canada currently? |  |
| Not serious | 21 (1.1) |
| Slightly serious | 94 (4.7) |
| Somewhat serious | 218 (10.9) |
| Moderately serious | 426 (21.2) |
| Very serious | 1236 (62.1) |
| Prefer not to answer | 4 (0.2) |
| How serious of a problem is COVID-19 in Canada compared to other countries in the world? |  |
| Much less | 116(5.8) |
| Slightly less | 799 (40.1) |
| About the same | 807 (40.6) |
| Slightly more | 170 (8.5) |
| Much more | 98 (4.9) |
| Prefer not to answer | 6 (0.3) |
| **Knowledge of COVID-19** |  |
| How do you rate your general understanding of how the virus is spread? |  |
| Poor | 35 (1.8) |
| Fair | 211 (10.6) |
| Good | 625 (31.14) |
| Very Good | 794 (39.9) |
| Excellent | 324 (16.3) |
| Prefer not to answer | 7 (0.4) |
| How often do you look for information related to COVID-19? |  |
| Never | 97 (4.9) |
| Once a week | 177 (8.9) |
| Every couple of days | 363 (18.3) |
| Once a day | 792 (40.0) |
| Several times a day | 553 (27.9) |
| Prefer not to answer | 14 (0.7) |
|  |  |

**Figure A:** Perceived psychological impact of COVID-19 and sufficiency of government response
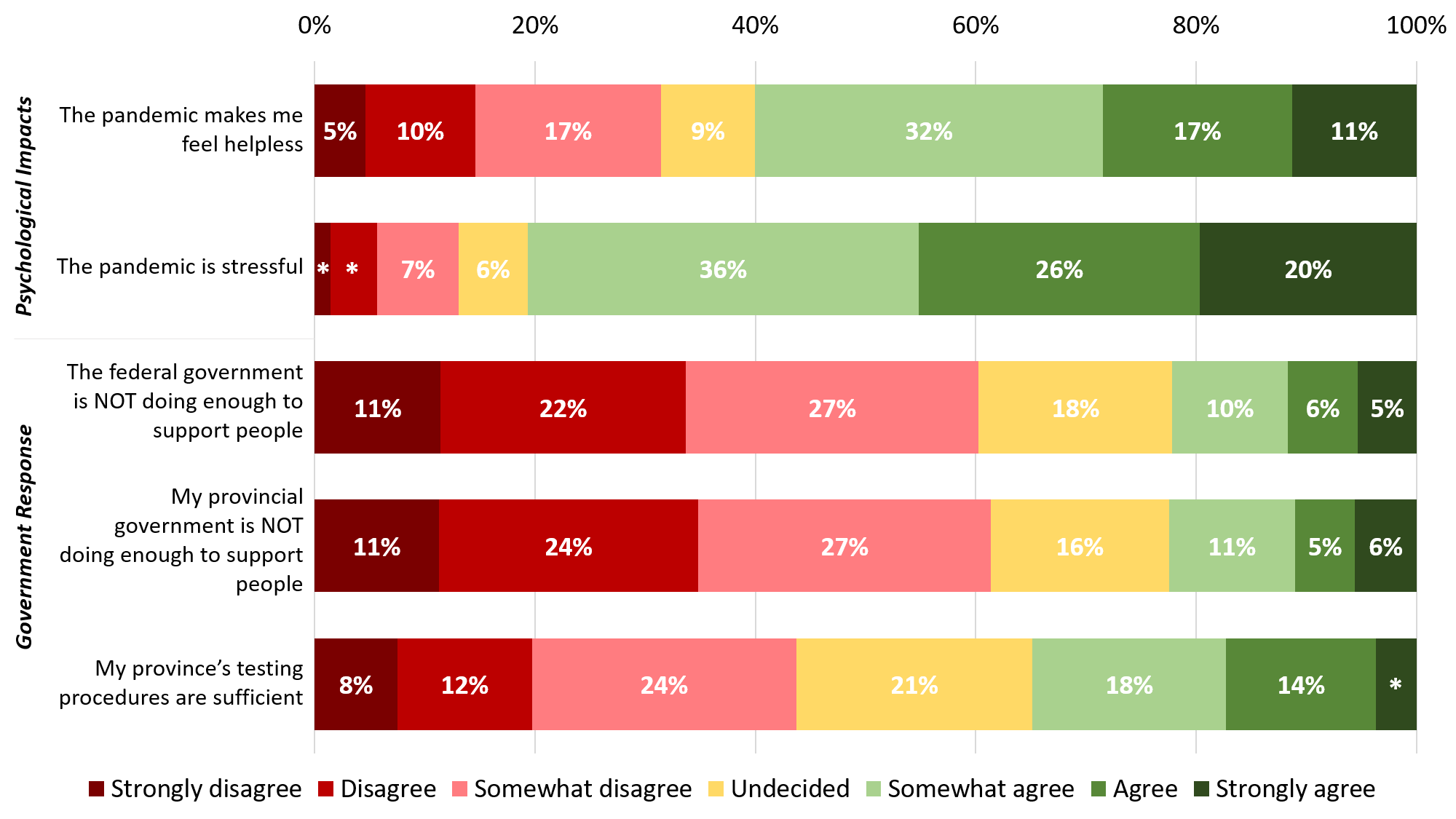


Note: Prefer not to answer response options are excluded from data analysis (range: n=7, 0.4% to n=95, 4.8%).

**Figure B.** Proportion of respondents who used various strategies to fact-check misinformation


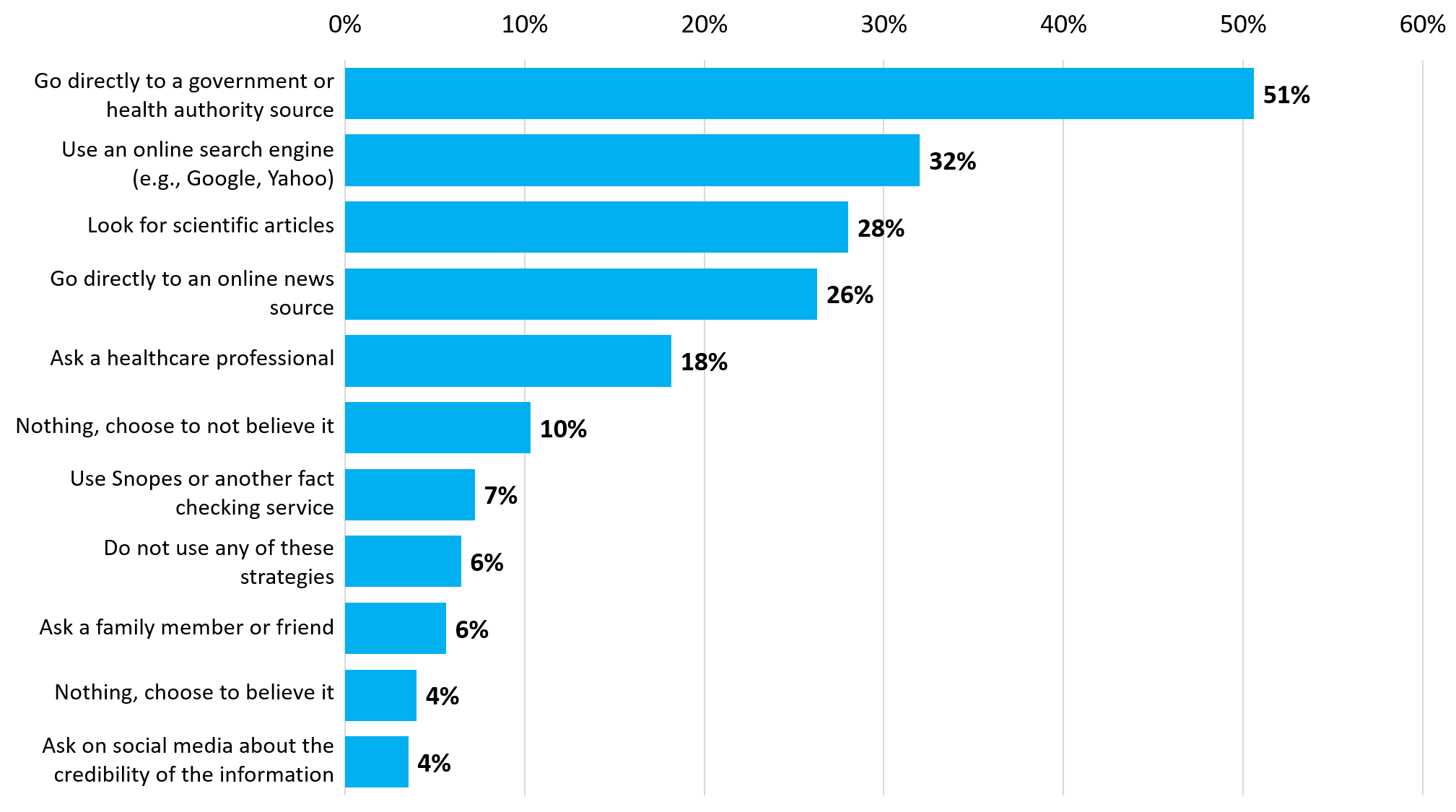


Notes: Respondents were asked to select all that apply, so responses add up to more than 100%. Prefer not to answer and “other” response options are excluded from data analyses (n=60, 3.0%).

**Figure C.** Respondents’ evaluation of information seeking and preventative behaviors.


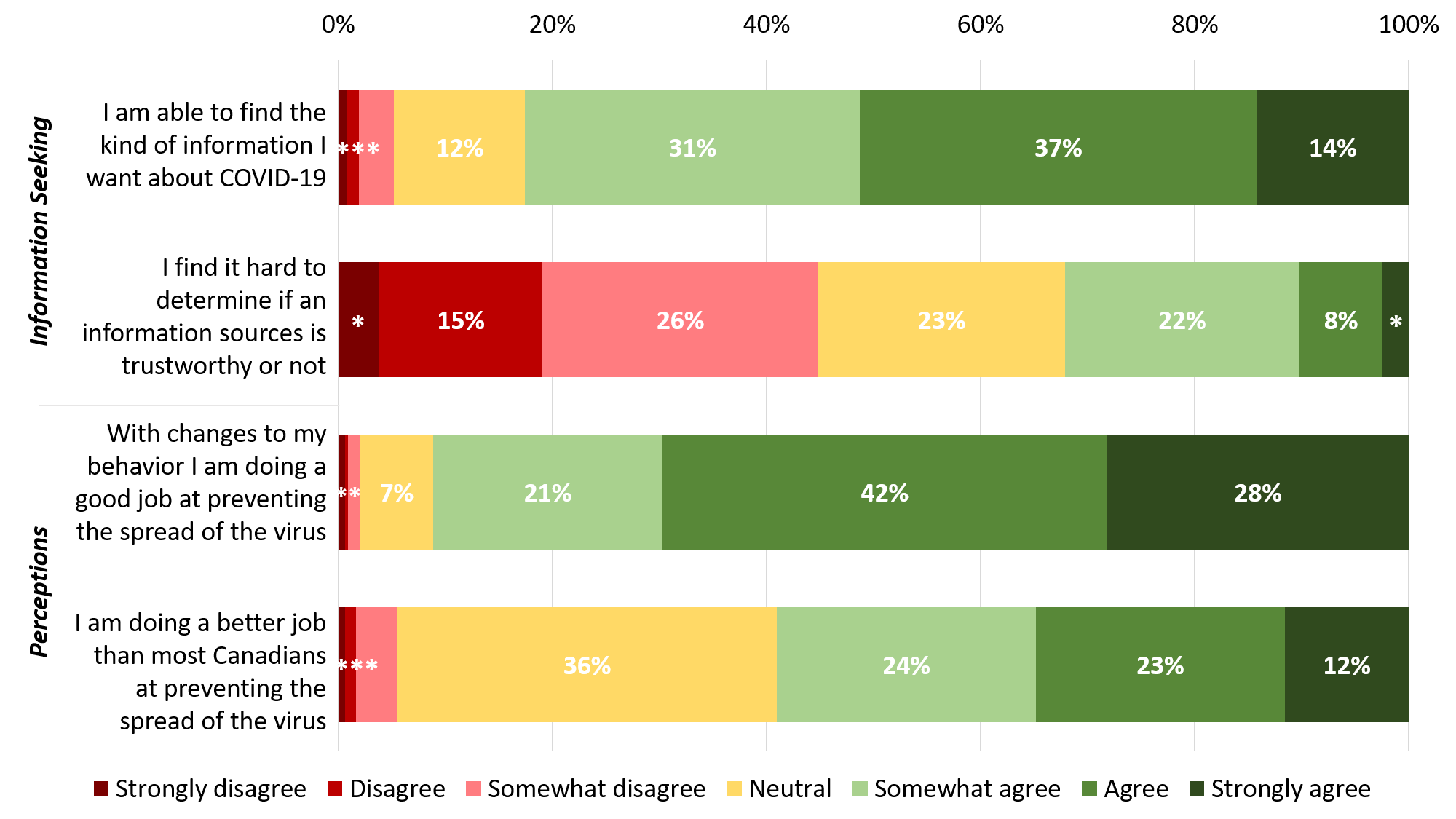


Notes: Prefer not to answer and “other” response options are excluded from data analysis (range: n=13, 0.7% to n=55 , 2.8%).

* = 1%

† = 2%

**Figure D.** Topics search for and topics identified as misinformation.


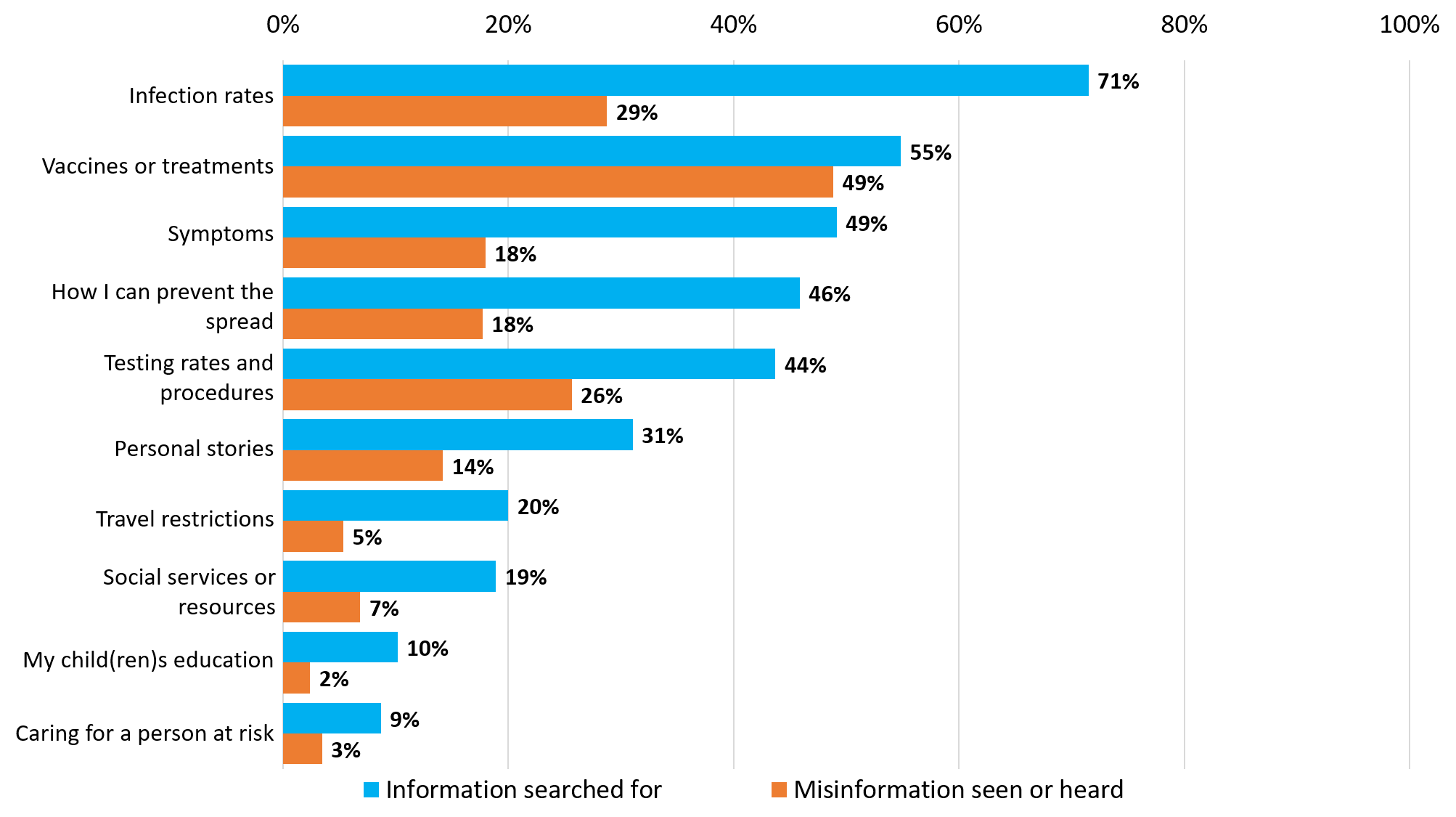


Note: Prefer not to answer response options are excluded from data analyses: “information searched for” (n=18, 0.9%), “misinformation seen or heard” (n=89, 4.5%).

**Figure E.** Degree of confidence in self and others ability to identify misinformation.


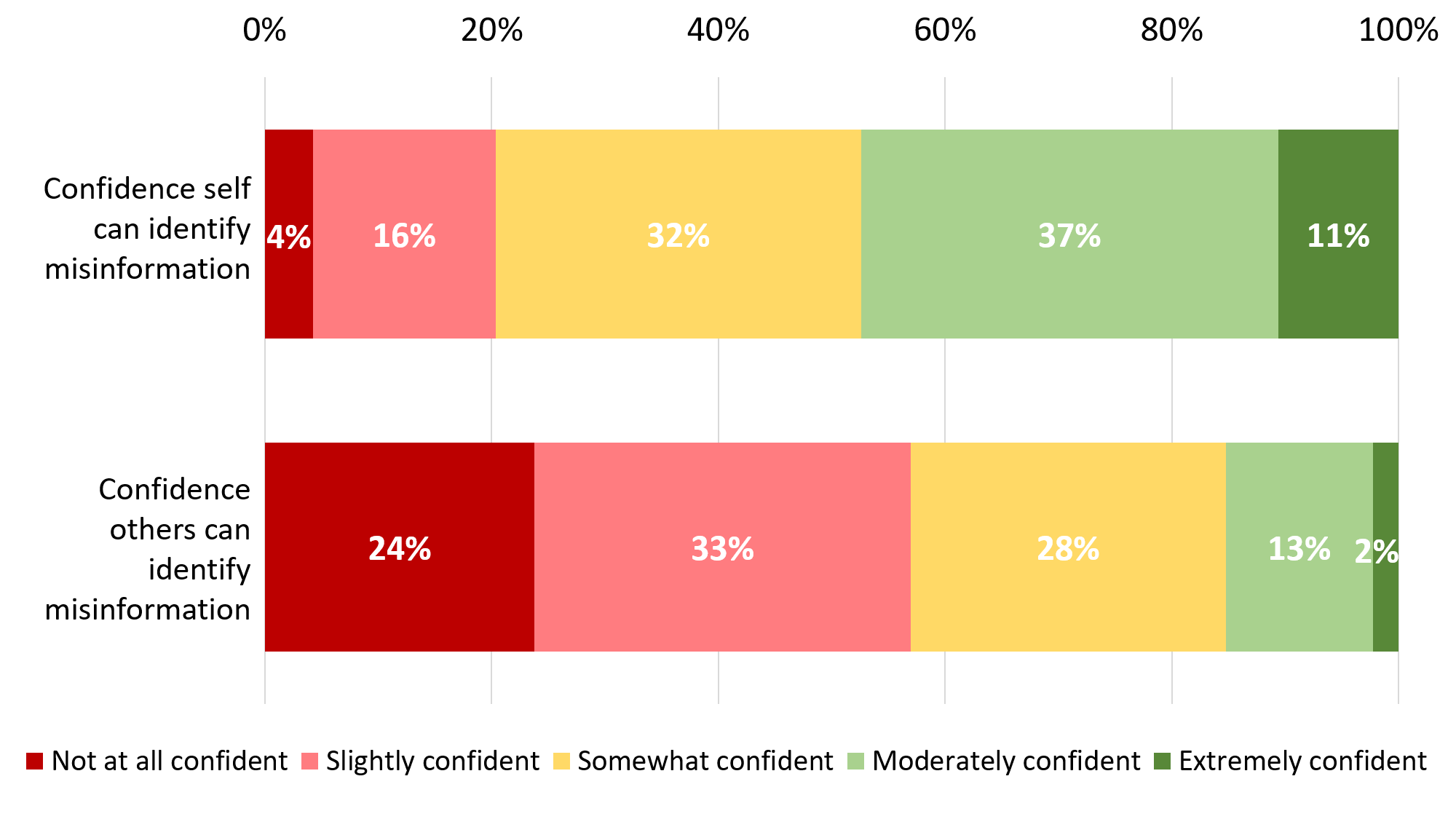


Note: Prefer not to answer response options are excluded from data analysis (n=19, 1.0% and n=24, 1.2%, respectively)

**Figure F.** Perceptions of age groups most and least consistently practicing physical distancing.


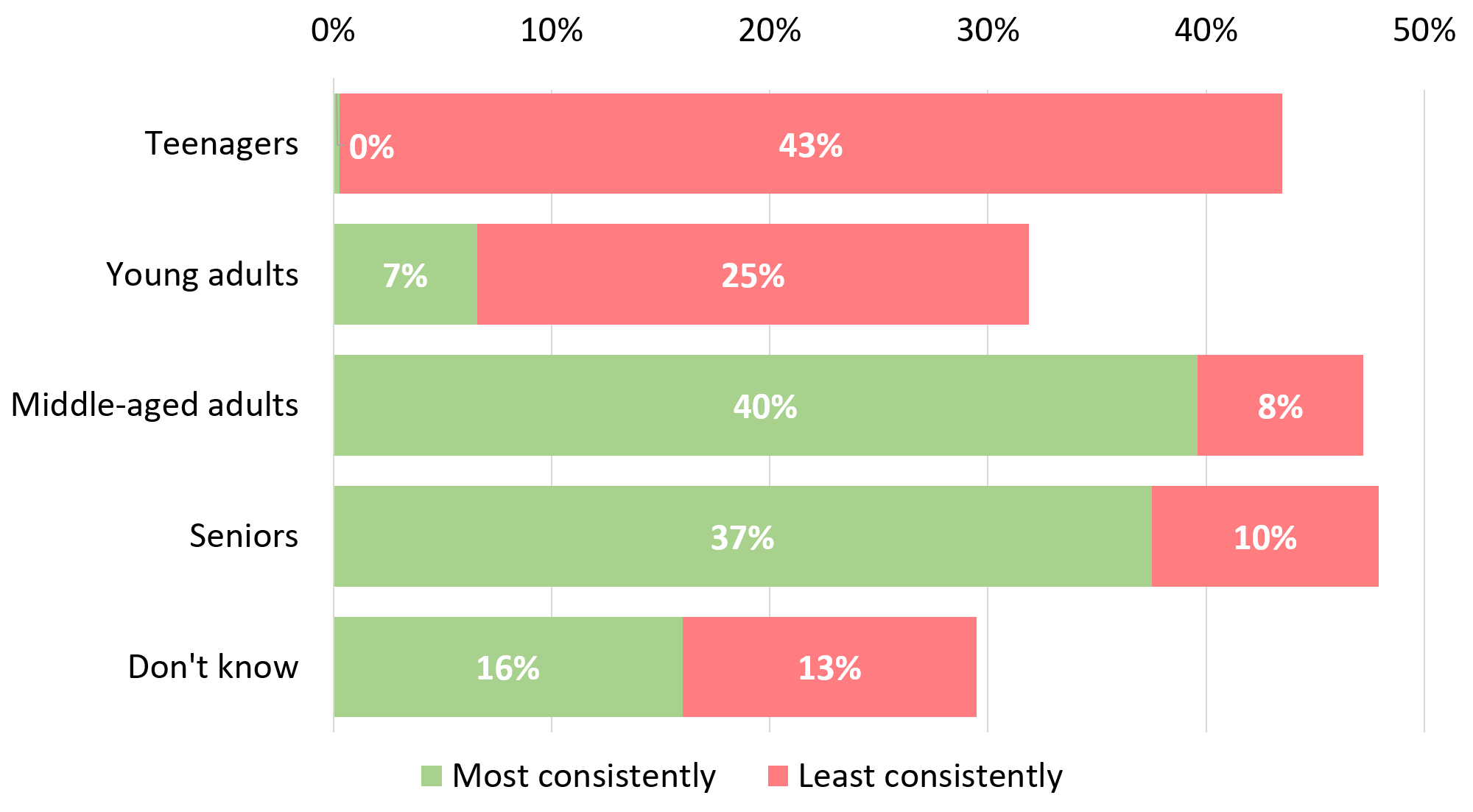


Note: Prefer not to answer response options are excluded from data analyses (n=11, 0.6% and n=17, 0.9%, respectively).

1. Weighted frequencies and percent are noted unless otherwise indicated. Prefer not to answer response options are excluded from data analyses. [↑](#footnote-ref-1)
