# Supplementary material for: A national cross-sectional survey of public perceptions of the COVID-19 pandemic: Self-reported beliefs, knowledge, and behaviors
Source: PLoS One. 2020 Oct 23;15(10):e0241259. doi: 10.1371/journal.pone.0241259 (PMC7584165; doi:10.1371/journal.pone.0241259)
Supplement: S3 Appendix — (DOCX) [file pone.0241259.s003.docx]

**S3 Appendix. Regional Differences in Self-Reported Perceptions**

Table A. Significant Chi-squared tests [2](#TableA)

Table B. Post-hoc Odds Ratios [5](#TableB)

**Table A. Significant Chi-squared tests of regional self-reported beliefs, knowledge acquisition, and behaviors related to COVID-19**

|  |  |  |  | **Regions (Weighted Percent)** | | | | | | **P-value** |
| --- | --- | --- | --- | --- | --- | --- | --- | --- | --- | --- |
|  |  |  |  | BC | AB | MN/SK | ON | QC | Atlantic |  |
|  |  | Do you know someone who has tested positive for COVID-19?^[[1]](#footnote-1)^ | Yes, close friends | 3.5 | 3.2 | 2.9 | 3.2 | 7.1 | 2.9 | 0.014 |
|  |  |  | Yes, family members not in same household | 3.5 | 4.1 | 1.4 | 4.9 | 7.5 | 5.0 | 0.048 |
| Beliefs | Severity | How serious of a problem is COVID-19 in Canada? | Not serious | 0.8 | 3.7 | 2.1 | 0.5 | 0.4 | 1.4 | <0.001 |
|  |  |  | Slightly serious | 3.5 | 6.8 | 7.8 | 4.1 | 4.4 | 5.7 |  |
|  |  |  | Somewhat serious | 8.9 | 13.7 | 13.5 | 7.4 | 15.9 | 10.0 |  |
|  |  |  | Moderately serious | 25.9 | 24.7 | 21.3 | 20.1 | 18.9 | 21.4 |  |
|  |  |  | Very serious | 61.0 | 51.1 | 55.3 | 67.9 | 60.4 | 61.4 |  |
|  |  | How serious of a problem is COVID-19 in Canada compared to other countries? | Much less serious | 6.6 | 11.4 | 10.0 | 4.0 | 4.6 | 5.0 | <0.001 |
|  |  |  | Slightly less serious | 52.3 | 44.3 | 50.7 | 41.0 | 27.7 | 38.6 |  |
|  |  |  | About the same | 34.1 | 36.5 | 29.3 | 38.4 | 52.4 | 42.1 |  |
|  |  |  | Slightly more serious | 3.5 | 4.1 | 5.7 | 11.9 | 8.6 | 9.3 |  |
|  |  |  | Much more serious | 3.5 | 3.7 | 4.3 | 4.8 | 6.7 | 5.0 |  |
|  | Concerns | I will contract COVID-19 | Not at all | 14.0 | 17.2 | 21.3 | 11.7 | 18.2 | 19.5 | 0.002 |
|  |  |  | Slightly | 39.5 | 36.7 | 31.6 | 31.2 | 23.5 | 29.3 |  |
|  |  |  | Somewhat | 20.2 | 20.9 | 18.4 | 24.7 | 25.1 | 25.6 |  |
|  |  |  | Moderately | 16.5 | 14.4 | 20.6 | 19.8 | 21.3 | 14.3 |  |
|  |  |  | Extremely | 9.9 | 10.7 | 8.1 | 12.6 | 12.0 | 11.3 |  |
|  |  | A family member will contract COVID-19 | Not at all | 9.9 | 11.9 | 11.5 | 6.0 | 13.4 | 9.4 | 0.043 |
|  |  |  | Slightly | 21.0 | 25.1 | 26.6 | 22.3 | 18.3 | 23.7 |  |
|  |  |  | Somewhat | 23.4 | 23.3 | 20.1 | 23.6 | 22.6 | 25.9 |  |
|  |  |  | Moderately | 26.6 | 21.0 | 20.1 | 23.6 | 23.7 | 19.4 |  |
|  |  |  | Extremely | 19.0 | 18.7 | 21.6 | 24.5 | 22.0 | 21.6 |  |
|  |  | People will not be able to access the healthcare they need because of COVID-19 restrictions | Not at all | 10.2 | 12.3 | 15.7 | 6.3 | 14.2 | 11.5 | <0.001 |
|  |  |  | Slightly | 23.8 | 25.6 | 22.1 | 15.4 | 14.4 | 19.4 |  |
|  |  |  | Somewhat | 30.1 | 21.9 | 21.4 | 24.2 | 25.2 | 25.2 |  |
|  |  |  | Moderately | 21.5 | 24.2 | 22.9 | 30.6 | 27.3 | 27.3 |  |
|  |  |  | Extremely | 14.5 | 16.0 | 17.9 | 23.4 | 18.9 | 16.5 |  |
|  |  | There will not be enough protective equipment (e.g. masks) for hospital staff to stay safe | Not at all | 10.9 | 11.4 | 8.6 | 4.9 | 10.6 | 9.4 | <0.001 |
|  |  |  | Slightly | 17.4 | 17.3 | 24.5 | 15.1 | 18.2 | 20.3 |  |
|  |  |  | Somewhat | 24.8 | 25.9 | 18.0 | 19.7 | 24.4 | 26.8 |  |
|  |  |  | Moderately | 26.7 | 24.1 | 23.0 | 27.9 | 27.1 | 18.8 |  |
|  |  |  | Extremely | 20.0 | 21.4 | 25.9 | 32.4 | 19.7 | 24.6 |  |
|  |  | There will not be enough hospital equipment (e.g. beds) to care for patients with COVID-19 | Not at all | 13.2 | 17.8 | 13.7 | 6.8 | 18.6 | 13.7 | <0.001 |
|  |  |  | Slightly | 26.5 | 22.8 | 23.0 | 21.0 | 18.4 | 25.9 |  |
|  |  |  | Somewhat | 31.1 | 25.6 | 25.9 | 22.9 | 22.7 | 23.0 |  |
|  |  |  | Moderately | 17.5 | 19.2 | 23.7 | 27.6 | 23.7 | 21.6 |  |
|  |  |  | Extremely | 11.7 | 14.6 | 13.7 | 21.7 | 16.5 | 15.8 |  |
|  | Health | The COVID-19 pandemic is very stressful | Strongly disagree | 1.56.2 | 3.711.9 | 2.19.3 | 0.84.1 | 1.14.0 | 0.75.7 | <0.001 |
|  |  |  | Disagree/ | 4.6 | 8.2 | 7.1 | 3.3 | 2.9 | 5.0 |  |
|  |  |  | Somewhat disagree | 10.8 | 6.8 | 9.3 | 5.6 | 9.1 | 3.6 |  |
|  |  |  | Neutral | 7.7 | 5.0 | 7.1 | 4.8 | 9.1 | 3.6 |  |
|  |  |  | Somewhat agree | 30.9 | 32.4 | 35.7 | 36.8 | 36.6 | 38.6 |  |
|  |  |  | Agree | 28.6 | 22.4 | 271 | 27 | 20.8 | 30.7 |  |
|  |  |  | Strongly agree | 15.8 | 21.5 | 11.4 | 21.8 | 20.4 | 17.9 |  |
|  |  | The COVID-19 pandemic makes me feel helpless | Strongly disagree | 5.8 | 7.7 | 5.0 | 3.9 | 3.8 | 2.9 | <0.001 |
|  |  |  | Disagree | 12.0 | 9.0 | 15.0 | 12.4 | 3.4 | 12.9 |  |
|  |  |  | Somewhat disagree | 20.8 | 17.6 | 21.4 | 19.4 | 8.2 | 19.3 |  |
|  |  |  | Neutral | 7.7 | 8.6 | 7.9 | 9.2 | 8.2 | 8.6 |  |
|  |  |  | Somewhat agree | 27.8 | 32.6 | 28.6 | 29.2 | 38.1 | 30.0 |  |
|  |  |  | Agree | 16.6 | 16.3 | 18.6 | 16.5 | 18.1 | 19.3 |  |
|  |  |  | Strongly agree | 9.3 | 8.1 | 3.6 | 9.6 | 20.2 | 7.1 |  |
| Knowledge Acquisition |  | How do you rate your general understanding of how the virus that causes COVID-19 is spread? | Poor | 0.4 | 1.4 | 0.0 | 1.6 | 4.0 | 0.7 | <0.001 |
|  |  |  | Fair | 10.5 | 8.2 | 5.0 | 10.2 | 14.9 | 9.3 |  |
|  |  |  | Good | 35.7 | 30.6 | 35.7 | 30.5 | 31.7 | 27.1 |  |
|  |  |  | Very good | 39.1 | 42.5 | 52.1 | 40.0 | 33.6 | 45.0 |  |
|  |  |  | Excellent | 14.3 | 17.4 | 7.1 | 17.7 | 15.8 | 17.9 |  |
|  |  | Sources accessed for information about COVID-19 over the last 2 weeks?^1^ | Canadian television, print and websites  (collapsed category) | 73.2 | 68.8 | 80.0 | 77.4 | 80.1 | 60.1 | <0.001 |
|  |  |  | American television, print and websites  (collapsed category) | 33.9 | 21.4 | 25.7 | 33.4 | 11.0 | 23.9 | <0.001 |
|  |  |  | International broadcasts, print, and websites | 19.7 | 17.7 | 11.4 | 24.1 | 12.3 | 15.9 | <0.001 |
| Behaviors |  | I will get vaccinated for the virus when it is developed | Strongly disagree | 2.0 | 9.1 | 3.0 | 5.1 | 6.7 | 3.0 | <0.001 |
|  |  |  | Disagree | 2.8 | 3.3 | 3.0 | 3.0 | 4.1 | 3.3 |  |
|  |  |  | Somewhat disagree | 1.2 | 1.4 | 2.3 | 2.4 | 3.3 | 3.0 |  |
|  |  |  | Neutral | 14.3 | 12.0 | 9.8 | 11.6 | 16.5 | 14.1 |  |
|  |  |  | Somewhat agree | 7.6 | 7.2 | 12.0 | 11.8 | 17.6 | 13.3 |  |
|  |  |  | Agree | 31.5 | 23.4 | 21.8 | 19.7 | 15 | 25.2 |  |
|  |  |  | Strongly agree | 40.6 | 46.5 | 48.1 | 46.4 | 36.9 | 38.5 |  |
|  |  | With changes to my behaviour, I am doing a good job at preventing the spread of the virus that causes COVID-19 | Strongly disagree | 0.8 | .09 | 0 | 0.7 | 0.6 | 0 | <0.001 |
|  |  |  | Disagree | 0 | 0.5 | 0 | 0.1 | 0.2 | 1.4 |  |
|  |  |  | Somewhat disagree | 0.8 | 0.9 | 0 | 0.8 | 1.7 | 2.2 |  |
|  |  |  | Neutral | 4.7 | 8.7 | 9.9 | 5.1 | 7.4 | 12.3 |  |
|  |  |  | Somewhat agree | 22.5 | 21.0 | 19.1 | 20.2 | 23.8 | 21.0 |  |
|  |  |  | Agree | 45.0 | 45.7 | 46.1 | 45.0 | 32.1 | 38.4 |  |
|  |  |  | Strongly agree | 26.4 | 22.4 | 24.8 | 28.2 | 34.2 | 24.6 |  |

Abbreviations: AB, Alberta; BC, British Columbia; MB, Manitoba; ON, Ontario; QC, Québec; SK, Saskatchewan

Table B. Post-hoc Odds Ratios

|  |  | **Odds Ratio (95% CI**) | | | | | **p-value** | | | | |
| --- | --- | --- | --- | --- | --- | --- | --- | --- | --- | --- | --- |
|  |  | BC vs ON | AB vs ON | SK/MB vs ON | QC vs ON | Atlantic vs ON | BC vs ON | AB vs ON | SK/MB vs ON | QC vs ON | Atlantic vs ON |
| Know someone who has tested positive for COVID-19? | Yes, close friends | 1.06 (0.48-2.33) | 0.96 (0.39-2.39) | 0.99 (0.33-2.92) | 2.39 (1.39-4.11) | 0.95 (0.32-2.80) | 0.88 | 0.93 | 0.98 | 0.002 | 0.93 |
|  | Yes, family members not in same household | 0.66 (0.31-1.39) | 0.81 (0.38-1.71) | 0.33 (0.08-1.39) | 1.57 (0.97-2.54) | 1.04 (0.45-2.39) | 0.27 | 0.58 | 0.13 | 0.065 | 0.93 |
| How serious of a problem is COVID-19 in Canada? | Not serious | 1.39 (0.25-7.79) | 6.47 (1.87-22.46) | 3.33 (0.60-18.44) | 0.93 (0.17-5.14) | 3.08 (0.56-17.09) | 0.71 | 0.003 | 0.17 | 0.94 | 0.2 |
|  | Slightly serious | 0.81 (0.38-1.74) | 1.73 (0.91-3.27) | 1.97 (0.94-4.14) | 1.09 (0.61-1.95) | 1.43 (0.64-3.20) | 0.59 | 0.094 | 0.074 | 0.76 | 0.38 |
|  | Somewhat serious | 1.24 (0.74-2.06) | 2.02 (1.25-3.25) | 1.91 (1.07-3.42) | 2.36 (1.62-3.43) | 1.36 (0.72-2.58) | 0.41 | 0.004 | 0.03 | <0.001 | 0.34 |
|  | Moderately serious | 1.38 (1.00-1.92) | 1.30 (0.91-1.86) | 1.10 (0.70-1.75) | 0.92 (0.69-1.24) | 1.10 (0.70-1.73) | 0.052 | 0.15 | 0.67 | 0.59 | 0.69 |
|  | Very serious | 0.74 (0.56-0.99) | 0.50 (0.37-0.67) | 0.59 (0.40-0.86) | 0.72 (0.57-0.92) | 0.74 (0.51-1.09) | 0.045 | <0.001 | 0.007 | 0.008 | 0.13 |
| How serious of a problem is COVID-19 in Canada compared to other countries? | Much less serious | 1.70 (0.93-3.10) | 3.14 (1.80-5.45) | 2.69 (1.36-5.32) | 1.16 (0.66-2.05) | 1.36 (0.58-3.16) | 0.084 | <0.001 | 0.004 | 0.61 | 0.48 |
|  | Slightly less serious | 1.58 (1.19-2.09) | 1.14 (0.84-1.54) | 1.52 (1.04-2.21) | 0.55 (0.43-0.71) | 0.92 (0.63-1.33) | 0.001 | 0.39 | 0.03 | <0.001 | 0.65 |
|  | About the same | 0.83 (0.62-1.11) | 0.92 (0.68-1.26) | 0.66 (0.44-1.00) | 1.76 (1.40-2.23) | 1.16 (0.80-1.68) | 0.21 | 0.62 | 0.049 | <0.001 | 0.45 |
|  | Slightly more serious | 0.27 (0.13-0.54) | 0.31 (0.15-0.63) | 0.42 (0.19-0.95) | 0.70 (0.47-1.05) | 0.75 (0.40-1.41) | <0.001 | 0.001 | 0.036 | 0.082 | 0.37 |
|  | Much more serious | 0.76 (0.37-1.56) | 0.78 (0.36-1.72) | 0.84 (0.32-2.18) | 1.45 (0.89-2.38) | 1.00 (0.41-2.45) | 0.45 | 0.54 | 0.71 | 0.14 | 0.99 |
| I will contract COVID-19 | Not at all | 1.24 (0.81-1.89) | 1.57 (1.03-2.39) | 2.07 (1.27-3.36) | 1.67 (1.19-2.34) | 1.80 (1.10-2.96) | 0.33 | 0.037 | 0.004 | 0.003 | 0.02 |
|  | Slightly | 1.43 (1.06-1.93) | 1.28 (0.93-1.77) | 1.01 (0.67-1.52) | 0.68 (0.52-0.89) | 0.93 (0.61-1.40) | 0.018 | 0.13 | 0.96 | 0.005 | 0.72 |
|  | Somewhat | 0.78 (0.55-1.10) | 0.81 (0.56-1.17) | 0.70 (0.43-1.12) | 1.02 (0.77-1.34) | 1.05 (0.68-1.62) | 0.16 | 0.26 | 0.14 | 0.91 | 0.82 |
|  | Moderately | 0.80 (0.55-1.17) | 0.68 (0.45-1.04) | 1.05 (0.66-1.69) | 1.10 (0.82-1.47) | 0.69 (0.40-1.17) | 0.25 | 0.074 | 0.83 | 0.53 | 0.17 |
|  | Extremely | 0.75 (0.47-1.20) | 0.82 (0.50-1.35) | 0.59 (0.30-1.17) | 0.95 (0.66-1.36) | 0.86 (0.47-1.56) | 0.23 | 0.44 | 0.13 | 0.78 | 0.61 |
| A family member will contract COVID-19 | Not at all | 1.67 (1.01-2.78) | 2.09 (1.25-3.48) | 1.98 (1.07-3.69) | 2.41 (1.60-3.61) | 1.65 (0.86-3.17) | 0.047 | 0.005 | 0.031 | <0.001 | 0.13 |
|  | Slightly | 0.93 (0.66-1.31) | 1.17 (0.83-1.66) | 1.27 (0.83-1.96) | 0.78 (0.58-1.05) | 1.07 (0.69-1.65) | 0.68 | 0.37 | 0.27 | 0.096 | 0.76 |
|  | Somewhat | 0.99 (0.71-1.38) | 0.97 (0.68-1.39) | 0.81 (0.51-1.29) | 0.95 (0.72-1.25) | 1.15 (0.76-1.75) | 0.98 | 0.88 | 0.37 | 0.71 | 0.51 |
|  | Moderately | 1.17 (0.85-1.62) | 0.87 (0.60-1.26) | 0.83 (0.52-1.32) | 1.01 (0.77-1.33) | 0.78 (0.49-1.24) | 0.34 | 0.46 | 0.44 | 0.94 | 0.29 |
|  | Extremely | 0.73 (0.52-1.03) | 0.71 (0.49-1.04) | 0.84 (0.54-1.33) | 0.86 (0.65-1.14) | 0.84 (0.54-1.31) | 0.076 | 0.076 | 0.47 | 0.3 | 0.44 |
| People will not be able to access the healthcare they need because of COVID-19 restrictions | Not at all | 1.72 (1.05-2.82) | 2.12 (1.28-3.49) | 2.84 (1.63-4.95) | 2.47 (1.66-3.67) | 1.89 (1.02-3.50) | 0.03 | 0.003 | <0.001 | <0.001 | 0.043 |
|  | Slightly | 1.69 (1.19-2.39) | 1.86 (1.30-2.66) | 1.52 (0.96-2.42) | 0.91 (0.66-1.27) | 1.34 (0.84-2.14) | 0.003 | 0.001 | 0.078 | 0.59 | 0.22 |
|  | Somewhat | 1.35 (0.99-1.85) | 0.88 (0.61-1.26) | 0.85 (0.54-1.34) | 1.06 (0.81-1.38) | 1.06 (0.69-1.62) | 0.057 | 0.48 | 0.49 | 0.69 | 0.79 |
|  | Moderately | 0.62 (0.45-0.87) | 0.73 (0.52-1.02) | 0.68 (0.44-1.05) | 0.85 (0.66-1.11) | 0.85 (0.56-1.28) | 0.005 | 0.069 | 0.081 | 0.23 | 0.43 |
|  | Extremely | 0.55 (0.37-0.80) | 0.62 (0.42-0.93) | 0.71 (0.44-1.15) | 0.76 (0.57-1.01) | 0.65 (0.40-1.06) | 0.002 | 0.019 | 0.16 | 0.063 | 0.083 |
| There will not be enough protective equipment for hospital staff to stay safe | Not at all | 2.32 (1.39-3.86) | 2.50 (1.47-4.24) | 1.86 (0.94-3.69) | 2.30 (1.47-3.59) | 2.07 (1.07-4.02) | 0.001 | 0.001 | 0.074 | <0.001 | 0.032 |
|  | Slightly | 1.19 (0.82-1.73) | 1.18 (0.79-1.77) | 1.84 (1.17-2.89) | 1.25 (0.92-1.71) | 1.40 (0.87-2.24) | 0.36 | 0.42 | 0.009 | 0.15 | 0.16 |
|  | Somewhat | 1.34 (0.96-1.87) | 1.43 (1.01-2.03) | 0.89 (0.55-1.44) | 1.31 (0.99-1.73) | 1.49 (0.98-2.28) | 0.083 | 0.046 | 0.64 | 0.062 | 0.065 |
|  | Moderately | 0.94 (0.69-1.30) | 0.81 (0.57-1.15) | 0.77 (0.50-1.20) | 0.96 (0.74-1.25) | 0.60 (0.38-0.95) | 0.73 | 0.24 | 0.25 | 0.77 | 0.029 |
|  | Extremely | 0.53 (0.38-0.74) | 0.57 (0.40-0.81) | 0.72 (0.47-1.10) | 0.51 (0.39-0.68) | 0.69 (0.45-1.05) | <0.001 | 0.002 | 0.13 | <0.001 | 0.086 |
| There will not be enough hospital equipment to care for patients with COVID-19 | Not at all | 2.07 (1.31-3.27) | 2.96 (1.89-4.64) | 2.20 (1.24-3.92) | 3.12 (2.16-4.52) | 2.16 (1.23-3.80) | 0.002 | <0.001 | 0.007 | <0.001 | 0.007 |
|  | Slightly | 1.36 (0.98-1.87) | 1.10 (0.77-1.58) | 1.12 (0.72-1.76) | 0.85 (0.63-1.14) | 1.31 (0.86-2.01) | 0.065 | 0.59 | 0.61 | 0.28 | 0.21 |
|  | Somewhat | 1.51 (1.11-2.07) | 1.15 (0.81-1.63) | 1.17 (0.76-1.80) | 0.99 (0.75-1.30) | 1.01 (0.65-1.56) | 0.009 | 0.42 | 0.48 | 0.93 | 0.96 |
|  | Moderately | 0.56 (0.39-0.79) | 0.63 (0.43-0.91) | 0.82 (0.53-1.27) | 0.82 (0.63-1.07) | 0.73 (0.47-1.14) | 0.001 | 0.014 | 0.37 | 0.15 | 0.17 |
|  | Extremely | 0.48 (0.32-0.73) | 0.62 (0.41-0.94) | 0.57 (0.33-0.97) | 0.71 (0.53-0.96) | 0.66 (0.40-1.10) | <0.001 | 0.024 | 0.037 | 0.027 | 0.11 |
| The COVID-19 pandemic is very stressful | Strongly disagree | 1.87 (0.52-6.72) | 4.64 (1.59-13.59) | 2.83 (0.69-11.53) | 1.27 (0.38-4.23) | 1.02 (0.12-8.56) | 0.34 | 0.005 | 0.15 | 0.69 | 0.99 |
|  | Disagree | 1.43 (0.70-2.92) | 2.66 (1.41-5.02) | 2.19 (0.99-4.85) | 0.90 (0.45-1.78) | 1.56 (0.65-3.71) | 0.32 | 0.003 | 0.053 | 0.76 | 0.32 |
|  | Somewhat disagree | 2.05 (1.25-3.36) | 1.26 (0.68-2.32) | 1.72 (0.88-3.37) | 1.66 (1.06-2.61) | 0.67 (0.26-1.73) | 0.005 | 0.47 | 0.12 | 0.027 | 0.41 |
|  | Neutral | 1.61 (0.92-2.84) | 1.04 (0.52-2.07) | 1.54 (0.72-3.28) | 1.96 (1.23-3.11) | 0.68 (0.26-1.78) | 0.098 | 0.92 | 0.26 | 0.005 | 0.44 |
|  | Somewhat agree | 0.77 (0.57-1.04) | 0.82 (0.60-1.13) | 0.96 (0.65-1.41) | 1.00 (0.79-1.27) | 1.08 (0.74-1.57) | 0.088 | 0.22 | 0.82 | 0.99 | 0.71 |
|  | Agree | 1.09 (0.80-1.48) | 0.79 (0.55-1.12) | 1.00 (0.66-1.52) | 0.71 (0.54-0.94) | 1.20 (0.80-1.79) | 0.59 | 0.18 | 0.999 | 0.016 | 0.38 |
|  | Strongly agree | 0.68 (0.47-0.98) | 0.97 (0.67-1.40) | 0.47 (0.27-0.84) | 0.92 (0.69-1.22) | 0.78 (0.49-1.25) | 0.039 | 0.87 | 0.01 | 0.58 | 0.31 |
| The COVID-19 pandemic makes me feel helpless | Strongly disagree | 1.54 (0.82-2.90) | 2.12 (1.15-3.91) | 1.38 (0.59-3.23) | 0.97 (0.53-1.80) | 0.74 (0.25-2.15) | 0.18 | 0.016 | 0.46 | 0.93 | 0.58 |
|  | Disagree | 0.96 (0.62-1.47) | 0.71 (0.42-1.18) | 1.23 (0.72-2.11) | 0.25 (0.15-0.43) | 1.04 (0.60-1.82) | 0.84 | 0.18 | 0.45 | <0.001 | 0.88 |
|  | Somewhat disagree | 1.09 (0.77-1.55) | 0.89 (0.60-1.32) | 1.15 (0.73-1.82) | 0.38 (0.26-0.55) | 1.01 (0.64-1.61) | 0.61 | 0.55 | 0.56 | <0.001 | 0.96 |
|  | Neutral | 0.84 (0.50-1.40) | 0.91 (0.53-1.55) | 0.83 (0.42-1.67) | 0.87 (0.57-1.33) | 0.95 (0.50-1.81) | 0.49 | 0.73 | 0.61 | 0.53 | 0.88 |
|  | Somewhat agree | 0.93 (0.69-1.27) | 1.19 (0.86-1.64) | 0.98 (0.64-1.48) | 1.49 (1.17-1.91) | 1.03 (0.69-1.55) | 0.66 | 0.3 | 0.91 | 0.001 | 0.87 |
|  | Agree | 1.02 (0.70-1.48) | 0.98 (0.65-1.46) | 1.14 (0.70-1.86) | 1.12 (0.82-1.52) | 1.22 (0.76-1.94) | 0.92 | 0.91 | 0.61 | 0.48 | 0.41 |
|  | Strongly agree | 0.95 (0.59-1.54) | 0.83 (0.48-1.43) | 0.35 (0.14-0.88) | 2.38 (1.71-3.32) | 0.69 (0.33-1.42) | 0.83 | 0.5 | 0.026 | <0.001 | 0.31 |
| Rating general understanding of how the virus that causes COVID-19 is spread? | Poor | Not modelled due to small numbers | | | | | Not modelled due to small numbers | | | | |
|  | Fair | 1.03 (0.65-1.63) | 0.80 (0.47-1.36) | 0.44 (0.19-1.04) | 1.53 (1.08-2.18) | 0.91 (0.49-1.70) | 0.91 | 0.41 | 0.061 | 0.017 | 0.78 |
|  | Good | 1.26 (0.94-1.69) | 1.00 (0.73-1.39) | 1.25 (0.84-1.86) | 1.06 (0.83-1.37) | 0.85 (0.56-1.29) | 0.13 | 0.98 | 0.26 | 0.63 | 0.44 |
|  | Very good | 0.97 (0.73-1.29) | 1.10 (0.82-1.50) | 1.65 (1.13-2.40) | 0.76 (0.60-0.97) | 1.22 (0.84-1.77) | 0.84 | 0.52 | 0.009 | 0.026 | 0.29 |
|  | Excellent | 0.77 (0.52-1.14) | 0.97 (0.66-1.44) | 0.37 (0.19-0.73) | 0.87 (0.64-1.19) | 1.00 (0.62-1.60) | 0.2 | 0.89 | 0.004 | 0.39 | 0.99 |
| Sources accessed for information about COVID-19 over the last 2 weeks? | Canadian television, print and websites (collapsed category) | 0.79 (0.58-1.10) | 0.65 (0.46-0.90) | 1.19 (0.75-1.90) | 1.19 (0.89-1.58) | 0.44 (0.30-0.65) | 0.16 | 0.011 | 0.46 | 0.25 | <0.001 |
|  | American television, print and websites  (collapsed category) | 1.01 (0.75-1.36) | 0.54 (0.38-0.78) | 0.69 (0.45-1.06) | 0.25 (0.18-0.34) | 0.62 (0.40-0.95) | 0.94 | 0.001 | 0.09 | <0.001 | 0.029 |
|  | International broadcasts, print, and websites | 0.77 (0.54-1.09) | 0.68 (0.46-1.00) | 0.40 (0.22-0.71) | 0.44 (0.32-0.61) | 0.60 (0.37-0.99) | 0.14 | 0.049 | 0.002 | <0.001 | 0.048 |
| I will get vaccinated for the virus when it is developed | Strongly disagree | 0.39 (0.15-1.00) | 1.91 (1.08-3.40) | 0.57 (0.20-1.64) | 1.33 (0.81-2.20) | 0.58 (0.20-1.67) | 0.049 | 0.027 | 0.3 | 0.26 | 0.31 |
|  | Disagree | 0.93 (0.39-2.24) | 1.11 (0.46-2.65) | 1.07 (0.36-3.20) | 1.41 (0.74-2.68) | 1.03 (0.35-3.06) | 0.88 | 0.82 | 0.9 | 0.3 | 0.96 |
|  | Somewhat disagree | 0.50 (0.14-1.72) | 0.56 (0.16-1.94) | 1.07 (0.31-3.72) | 1.33 (0.65-2.71) | 1.33 (0.44-4.04) | 0.27 | 0.36 | 0.92 | 0.44 | 0.61 |
|  | Neutral | 1.27 (0.83-1.93) | 1.03 (0.64-1.66) | 0.83 (0.44-1.57) | 1.50 (1.07-2.11) | 1.25 (0.73-2.14) | 0.27 | 0.9 | 0.57 | 0.02 | 0.42 |
|  | Somewhat agree | 0.60 (0.36-1.02) | 0.58 (0.33-1.02) | 1.03 (0.57-1.85) | 1.60 (1.14-2.23) | 1.14 (0.65-2.00) | 0.06 | 0.061 | 0.93 | 0.006 | 0.65 |
|  | Agree | 1.87 (1.36-2.58) | 1.24 (0.86-1.80) | 1.12 (0.70-1.79) | 0.71 (0.52-0.98) | 1.36 (0.88-2.11) | <0.001 | 0.25 | 0.64 | 0.04 | 0.17 |
|  | Strongly agree | 0.79 (0.59-1.06) | 0.89 (0.65-1.21) | 1.06 (0.72-1.56) | 0.68 (0.53-0.87) | 0.72 (0.49-1.05) | 0.11 | 0.46 | 0.77 | 0.002 | 0.091 |
|  | Strongly disagree | Not modelled due to small numbers | | | | | Not modelled due to small numbers | | | | |
| With changes to my behaviour, I am doing a good job at preventing the spread of the virus that causes COVID-19 | Disagree | Not modelled due to small numbers | | | | | Not modelled due to small numbers | | | | |
|  | Somewhat disagree | Not modelled due to small numbers | | | | | Not modelled due to small numbers | | | | |
|  | Neutral | 0.95 (0.49-1.82) | 1.77 (1.00-3.12) | 2.07 (1.05-4.09) | 1.50 (0.92-2.44) | 2.68 (1.44-4.99) | 0.88 | 0.05 | 0.037 | 0.1 | 0.002 |
|  | Somewhat agree | 1.16 (0.83-1.63) | 1.06 (0.73-1.53) | 0.94 (0.58-1.52) | 1.24 (0.94-1.65) | 1.04 (0.66-1.64) | 0.39 | 0.78 | 0.8 | 0.13 | 0.87 |
|  | Agree | 1.00 (0.75-1.32) | 1.02 (0.76-1.38) | 1.05 (0.72-1.53) | 0.58 (0.45-0.74) | 0.75 (0.52-1.10) | 0.99 | 0.89 | 0.78 | <0.001 | 0.14 |
|  | Strongly agree | 0.90 (0.66-1.24) | 0.74 (0.52-1.06) | 0.83 (0.55-1.28) | 1.31 (1.02-1.69) | 0.83 (0.54-1.26) | 0.52 | 0.1 | 0.4 | 0.032 | 0.38 |

Abbreviations: AB, Alberta; BC, British Columbia; CI, Confidence Interval; MB, Manitoba; ON, Ontario; QC, Québec; SK, Saskatchewan

1. Question format is multiple response checkbox. Only response item with significant Chi-square results are presented. Percentages will not add up to 100. [↑](#footnote-ref-1)
