# Supplementary material for: A national cross-sectional survey of public perceptions of the COVID-19 pandemic: Self-reported beliefs, knowledge, and behaviors
Source: PLoS One. 2020 Oct 23;15(10):e0241259. doi: 10.1371/journal.pone.0241259 (PMC7584165; doi:10.1371/journal.pone.0241259)
Supplement: S1 Fig — (DOCX) [file pone.0241259.s004.docx]

**S1 Fig. Survey content domains of public perceptions.**
